# Evaluation of the Production of Dissolved Organic Matter by Three Marine Bacterial Strains

**DOI:** 10.3389/fmicb.2020.584419

**Published:** 2020-10-15

**Authors:** Shuji Goto, Yuya Tada, Koji Suzuki, Youhei Yamashita

**Affiliations:** ^1^Graduate School of Environmental Science, Hokkaido University, Sapporo, Japan; ^2^National Institute for Minamata Disease, Minamata, Japan; ^3^Faculty of Environmental Earth Science, Hokkaido University, Sapporo, Japan

**Keywords:** microbial carbon pump, DOC, fluorescent DOM, *Alteromonas macleodii*, *Vibrio splendidus*, *Phaeobacter gallaeciensis*

## Abstract

A large part of marine dissolved organic matter (DOM) is considered to be recalcitrant DOM (RDOM) produced by marine bacteria. However, it is still unclear whether differences in bacterial species and/or physiology control the efficiency of RDOM production. Here, batch culture experiments with glucose as the sole carbon source were carried out using three model marine bacterial strains, namely, *Alteromonas macleodii* (Alt), *Vibrio splendidus* (Vib), and *Phaeobacter gallaeciensis* (Pha). Dissolved organic carbon (DOC) concentrations drastically decreased during the exponential growth phases of these bacteria due to the consumption of glucose. The efficiency of bacterial DOC production at the end of incubation was largely different among the strains and was higher for Vib (20%) than for the other two strains (Alt, 4%; Pha, 6%). All strains produced fluorescent DOM (FDOM), including humic-like FDOM which is considered as recalcitrant component in the ocean, even though the composition of bacterial FDOM was also different among the strains. The efficiency of humic-like FDOM production during the exponential growth phase was different among the bacterial strains; that is, Pha produced humic-like FDOM efficiently compared with the other two species. The efficiency of humic-like FDOM production with mineralization of organic matter was lower during the exponential growth phase than during the stationary phase of Alt and Pha. Four processes for the production of bacterially derived recalcitrant humic-like FDOM are suggested from this study: (1) production during active growing (in all strains), (2) production with the reutilization of bacterial DOM (Alt), (3) production with the consumption of cellular materials (Pha), and (4) release from lysis (Vib). Our results suggest that bacterial species and physiology can regulate RDOM production and accumulation in the ocean.

## Introduction

As a constituent of the reduced carbon pool, dissolved organic matter (DOM) in the ocean plays an important role in the global carbon cycle and is comparable in quantity to the atmospheric CO_2_ pool (Hedges, 1992; Hansell and Carlson, 1998; Hansell et al., 2009). The average radiocarbon age of bulk dissolved organic carbon (DOC) in the ocean has been estimated to be approximately 2,000–6,000 years (Bauer et al., 1992; Druffel et al., 1992; Beaupré, 2015). A physical/biogeochemical model of the global distribution of DOC in the ocean determined that the recalcitrant fraction, the lifetime of which is estimated to be approximately 15,000 years, contributes more than 90% of the bulk DOC (Hansell et al., 2009). Despite the importance of marine recalcitrant DOM (RDOM), as a slow cycling component in the global carbon cycle, the production mechanisms and/or sources of RDOM have not been well documented.

Cell wall polymers of marine microbes have been considered one of the major components of RDOM (Tanoue et al., 1995; McCarthy et al., 1998; Benner and Kaiser, 2003; Wakeham et al., 2003). Recently, it has also been suggested that degraded products of pigments (carotenoids and phycobilins) can become biogenic RDOM in the ocean (Arakawa et al., 2017; Zhao et al., 2017). The microbial production of DOM, which is resistant to microbial degradation, from labile substrates (e.g., glucose and glutamate) has been observed by *in vitro* incubation for between 20 days and 2 years with microbial communities from seawater (Brophy and Carlson, 1989; Ogawa et al., 2001; Kramer and Herndl, 2004; Kawasaki and Benner, 2006; Lønborg et al., 2009; Shimotori et al., 2009; Koch et al., 2014). The chemical composition, i.e., the *C*/*N* ratio, composition of amino acids and amino sugars, proportion of uncharacterized DOM fraction (Ogawa et al., 2001), fluorescent characteristics (Shimotori et al., 2009), and molecular pattern (determined by ultra-high-resolution mass spectrometry) (Koch et al., 2014; Lechtenfeld et al., 2015) of the microbially produced DOM was similar to that of the RDOM that occurs in marine environments, although it has also been reported that molecular composition of most microbial DOM is distinct from that of marine RDOM (Osterholz et al., 2015). In addition, the microbial production of humic-like fluorescent DOM (FDOM), which could be recalcitrant for centuries, has been highlighted for its basin-scale distributions (Yamashita and Tanoue, 2008; Catalá et al., 2015). Consequently, the microbial processes contributing marine RDOM formation has been termed “microbial carbon pump (MCP)”, which can contribute carbon sequestration in the ocean (Jiao et al., 2010).

It has been reported that the chemical composition of bacterially derived DOM is related to bacterial community composition (Lucas et al., 2016; Osterholz et al., 2016; Tada and Suzuki, 2016; Tada et al., 2017). A strong linkage between specific bacterial taxa and particular DOM molecules was found from the monitoring of DOM molecular composition determined by ultra-high-resolution mass spectrometry and bacterial community structure determined by 16S rRNA gene sequencing during phytoplankton blooms in the North Sea (Lucas et al., 2016). Such recent results obtained by state-of-the-art techniques suggest that some specific species and/or physiological states of marine bacteria control the efficiency of the MCP in the ocean. However, it is difficult to identify the key bacterial species and the physiological states that contribute to RDOM production from observations or *in vitro* experiments with bacterial communities.

Batch culture experiments using bacterial isolates could allow the evaluation of possible key bacterial species that greatly contribute to the MCP. Two previous studies incubated two *Roseobacter* species, *Phaeobacter inhibens* and *Dinoroseobacter shibae*, with simple substrates (glucose, glutamate, acetate or succinate) as the sole carbon source and found that the chemical composition of exometabolites was different between the two bacterial strains (Wienhausen et al., 2017; Noriega-Ortega et al., 2019). The results of these previous studies suggest that the capability for RDOM production is different among bacterial species. Furthermore, it was reported that an *Alteromonas* sp. strain, which was isolated from coastal seawater and shared ∼99% 16S ribosomal DNA sequence similarity with *Alteromonas macleodii*, had the capacity of alteration of marine DOM composition (Pedler et al., 2014; Pedler Sherwood et al., 2015). The previous studies (Pedler et al., 2014; Pedler Sherwood et al., 2015) suggested the presence of key bacterial species in carbon cycling including the MCP. Thus, comparison of the efficiency and processes of RDOM production among bacterial strains can provide new insights into the mechanisms of RDOM production and the MCP.

In this study, to investigate the differences in the quantity and quality of bacterial DOM among bacterial species and the key physiological processes for bacterial DOM production, *in vitro* incubations of model bacterial strains with glucose as a labile substrate were carried out. For the model bacterial strains, the gammaproteobacteria *Vibrio splendidus* and *Phaeobacter gallaeciensis*, which also belong to the Roseobacter clade of the alphaproteobacteria, were used. The microbial production of DOM as well as the recalcitrant humic-like FDOM production were measured. The results previously obtained by *in vitro* incubation with *A. macleodii* (Goto et al., 2017) were compared with those obtained from the two strains (*V. splendidus* and *P. gallaeciensis*) used in this study.

## Materials and Methods

### Marine Bacterial Strains

*V. splendidus* ATCC 25914 (Vib) and *P. gallaeciensis* ATCC 700781 (Pha) were obtained from the Japan Collection of Microorganisms, RIKEN BioResource Center (Tukuba, Ibaraki, Japan), and were used as model marine bacterial isolates in the present study. The previously reported results of *A. macleodii* (Alt) (Goto et al., 2017) were used for a comparison with those of Vib and Pha.

*A. macleodii* and *V. splendidus* belong to the ubiquitous gammaproteobacteria. *V. splendidus* has been reported to be a common heterotrophic, free-living bacteria in culturable bacterial communities in coastal and open oceans (Urakawa et al., 1999; Radjasa et al., 2001). The populations of *V. splendidus* in coastal regions have been reported to comprise variable phylogenetic groups adapted to differing ecological conditions, namely, seasons and habitats, and are categorized by their size fractions in samples (Hunt et al., 2008). These studies suggest that these strains are suitable model organisms of free-living marine bacteria and bacteria that respond to the supply of labile DOM.

*P. gallaeciensis* was used as the other model marine bacterial strain in this study. The genus *Phaeobacter*, belonging to the *Roseobacter* clade, is frequently predominant in phytoplankton blooms and has a versatile metabolism (Pommier et al., 2005; Newton et al., 2010). *P. gallaeciensis* has been reported to attach to zooplankton or coccolithophores (Seyedsayamdost et al., 2011; Freese et al., 2017). Therefore, the Pha strain is considered an appropriate model species of attached bacteria belonging to the *Roseobacter* clade.

Frozen stock of each bacterial strain was inoculated and then incubated in modified artificial seawater-based Aquil medium supplemented with glucose (1,000 μmol C L^–1^) at 25°C according to Goto et al. (2017). DOC concentration of the modified artificial seawater-based Aquil medium was 9.7 ± 2.0 μmol C L^–1^. The incubation periods were determined based on the lengths of the exponential growth phases of the three strains (24 h for Alt and Vib, 36 h for Pha). After each incubation was repeated twice, each medium was used as inoculum for the experiments described in the next section.

### Experimental Setup

The experimental design was basically the same as that described previously (Goto et al., 2017). The modified artificial seawater-based Aquil medium was used for each incubation. The nitrogen source for the Alt incubation was NaNO_3_ (Goto et al., 2017), while NH_4_Cl (161 μmol N L^–1^) was used for the incubations of Vib and Pha according to the nitrogen availability of these strains. The phosphorus source was NaH_2_PO_4_⋅H_2_O (10 μmol P L^–1^). Glucose (1,000 μmol C L^–1^) was added to the artificial seawater in the experimental treatments, while the water in the control treatments were prepared without glucose. The inoculum of each strain was added to the artificial seawater in each treatment at a dilution of 1:1000. Each bacterial strain was incubated in 100 mL of artificial seawater in acid-washed 250 mL polyethylene terephthalate bottles in the dark at 25°C. Triplicate bottles in the experimental and control treatments of each bacterial strain were sampled at the time points summarized in Supplementary Table 1 to determine the bacterial abundance, organic carbon concentration and DOM optical properties.

### Bacterial Abundance

Samples for the analyses of the bacterial abundance were fixed in paraformaldehyde [2% (vol/vol) final concentration] and preserved at −80°C. The bacterial cell density of the Vib treatment was measured with an EPICS flow cytometer (XL ADC system, Beckman Coulter) equipped with a 15 mW air-cooled laser excited at 488 nm, according to the protocol of Tada and Suzuki (2016). This method has been described in detail elsewhere (Goto et al., 2017). The bacterial abundance of the Pha treatment was measured with an epifluorescence microscope (BZ-9000, KEYENCE), since the cells possibly aggregated during incubation. The fixed samples were filtered onto polycarbonate membrane filters with a pore size of 0.2 μm (WHA-110656, Whatman, GE Healthcare) and strained with a DAPI mixture. The DAPI mixture constituted 5.5 parts (by volume) of Citifluor (Citifluor Ltd.), 1 part Vectashield (Vector Labs) and 0.5 parts PBS with 4′,6-diamidino-2-phenylindole at a final concentration of 2 μg mL^–1^. The filters were prepared for microscopic observation with glass slides and covers. Fifteen fluorescence microscopy images were stored as TIFF files per sample and analyzed by ImageJ software (ver. 1.49, Wayne Rasband, National Institutes of Health). The images were processed by the Laplace filter (5 × 5 kernel), Gaussian filter (radius 3) and median filter (rank 3) to define cell boundaries and remove noise (Fazi et al., 2008). After the procedures, the particles in each image were counted with the software.

### Organic Carbon Concentrations and DOM Optical Properties

Analytical procedures to determine the DOC concentration and DOM optical properties have been reported elsewhere (Goto et al., 2017) and are only briefly described here. The incubated media were filtered through precombusted (450°C, 3 h) glass fiber filters with a nominal pore size of 0.3 μm (GF75, Advantec) under gentle vacuum (<0.02 MPa) to measure the DOC concentration and DOM optical properties. The total organic carbon (TOC) concentration was measured in unfiltered samples, and the particulate organic carbon (POC) concentration, corresponding to the bacterial biomass possibly including the dead cells during the stationary and death phases, was calculated by subtracting the DOC concentration from the TOC concentration. Furthermore, we determined bacterial growth efficiency (BGE) at the initiation of the stationary phases. BGE is the ratio of bacterial biomass production (BP) to carbon demand, which is sum of bacterial biomass production and respiration (BR) (del Giorgio and Cole, 1998). In this study, the POC concentration and the decrease in TOC concentration during incubation were used for BP and BR, respectively.

The organic carbon concentrations, namely, the TOC and DOC concentrations, were determined by high-temperature catalytic oxidation with a total organic carbon analyzer (TOC-V CSH, Shimadzu). The accuracy and consistency of the measured organic carbon concentrations were checked by deep seawater consensus reference material (Hansell laboratory, University of Miami), which was assessed daily. The excitation-emission matrix (EEM) was measured using a fluorometer (FluoroMax-4, Horiba) according to the procedure of Tanaka et al. (2014). Several postacquisition steps were involved in the correction of the EEM, including instrumental bias correction, corrections of the inner filter effect using absorbance, and the subtraction of a blank (Milli-Q water) from the EEM, and fluorescence intensity in the EEM was converted to Raman Units (RUs), with the peak area of Raman scatter at 350 nm excitation (Lawaetz and Stedmon, 2009). The absorbance spectrum of each sample for correction of the inner filter effect was measured with a Shimadzu UV-1800 spectrophotometer in a 1-cm quartz cuvette according to Yamashita et al. (2013).

### Statistical Analysis

The contours of the EEMs were plotted with the statistical software R (version 3.2.3) (R Development Core Team, 2015). The linear regression analysis between the organic carbon concentration or the fluorescence intensity of the protein-like FDOM and the fluorescence intensity of the humic-like FDOM was performed with R to investigate the physiological processes of humic-like FDOM production.

## Results

The spectral characteristics of FDOM and their changes over time with bacterial abundance, DOC concentration and fluorescence intensity of each fluorophore in the Alt treatment were reported previously (Goto et al., 2017); these results were compared to those of Vib and Pha in this study. The TOC and POC concentrations in the Alt treatment are reported, for the first time, in this study.

### Growth of Bacteria and Change in Organic Carbon Concentration

**FIGURE 1:**
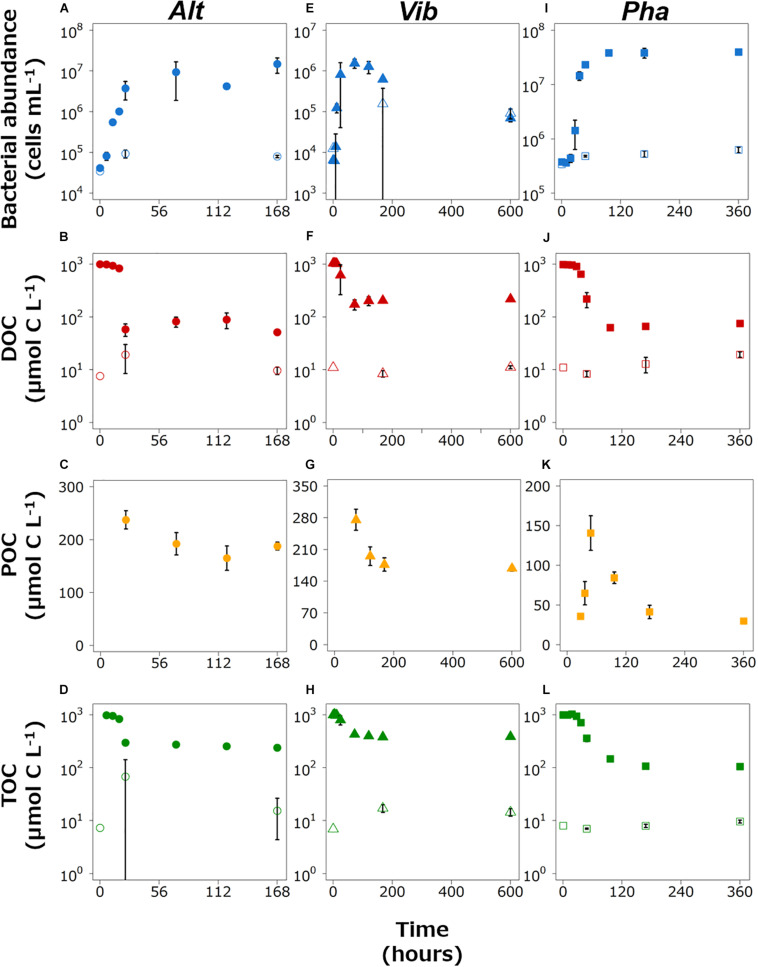
Changes in the bacterial abundance and the concentrations of dissolved organic carbon (DOC), particulate organic matter (POC), and total organic carbon (TOC) in the Alt **(A–D)**, Vib **(E–H)**, and Pha **(I–L)** treatments, respectively. The closed and open symbols represent the experimental treatment and control treatment, respectively. The error bars indicate standard deviation of triplicate incubation bottles.

The abundance of Alt in the experimental treatment increased exponentially during the period of 0–24 h and was stable from 24 h to the end of incubation (Figure 1A). The cell density in the control treatment increased slightly. The DOC concentration gradually decreased during the period of 0–18 h and then drastically decreased from 18 to 24 h of incubation (Figure 1B). The DOC concentrations were relatively stable during the period of 24–168 h. The DOC concentrations in the control treatment were quite low compared with the experimental treatment and did not change during the incubations. The POC concentration could not be determined during the first 24 h of incubation in the experimental treatment or throughout the incubation in the control treatment since the concentration was equivalent to the analytical errors of the DOC and TOC (Figure 1C). The POC concentration was above the detection limit at 24 h and fluctuated during the period of 24–168 h. The period from 0–18 h was defined as the exponential growth phase because the cell density did not reach its highest level and the DOC concentration, which was mainly derived from glucose, was still high during this period. The period from 24–168 h was defined as the stationary phase due to the stability of the cell density.

The abundance of Vib in the experimental treatment increased drastically just after the start of incubation and reached the highest cell density at 72 h (Figure 1E). Then, the abundance of Vib decreased until the end of incubation in the experimental treatment. In the control treatment, the abundance of Vib slightly increased from the initiation of incubation to other time points. The DOC concentration in the experimental treatment slightly decreased from the initial experiment to 12 h, largely decreased from 12 to 72 h, and gradually increased from 72 h to the end of incubation (Figure 1F). The DOC concentration in the control treatment was stable, with a relatively low concentration compared to that in the experimental treatment. The POC concentration in the experimental treatment was above the detection limit at 72 h (Figure 1G) and continued to decrease after 72 h of incubation in the experimental treatment. The POC concentration in the control treatment could not be determined throughout the incubation period. The period from 0–18 h was defined as the exponential growth phase, while the period from 72–600 h was defined as the stationary phase (including a part of the death phase) based on the changes over time in cell density and DOC concentration in the Vib experimental treatment. The time point of 24 h in the incubation was excluded from the definition of the growth phase because the triplicate bottles showed wide variability due to the boundary between the two growth phases.

The abundance of Pha in the experimental treatment increased exponentially from the initiation of incubation to 48 h, and the cell density remained at a higher level from 96 h to the end of incubation (Figure 1I). The cell concentrations in the control treatment did not change largely throughout the incubation. The DOC concentration in the experimental treatment decreased gradually from the initiation of incubation to 27 h and then decreased drastically to 96 h (Figure 1J). From 96 h to the end of incubation, the DOC concentration remained relatively low. The DOC concentrations in the control treatment were much lower than those in the experimental treatment throughout the incubation. The POC concentration was above the detection limit at 27 h, increased from 27 h by 48 h, and then decreased to the end of the incubation period (Figure 1K). Therefore, the period from 0–48 h was defined as the exponential growth phase, as the cell abundance increased in the period from 27–48 h. The cell abundance and DOC concentration were rather stable in period from 96–360 h; thus, this period was defined as the stationary phase.

The TOC concentration in the experimental treatment slightly decreased from the initial experiment, then sharply decreased just before the end of the exponential growth phase, and slightly decreased during the stationary phase for three strains incubations (Figures 1D,H,L). The BGEs at the initiation of the stationary phase in the experimental treatment of Alt, Vib, and Pha were 25, 33, and 9%, respectively.

### Spectral Characteristics of FDOM Produced by the Bacterial Strains

**FIGURE 2:**
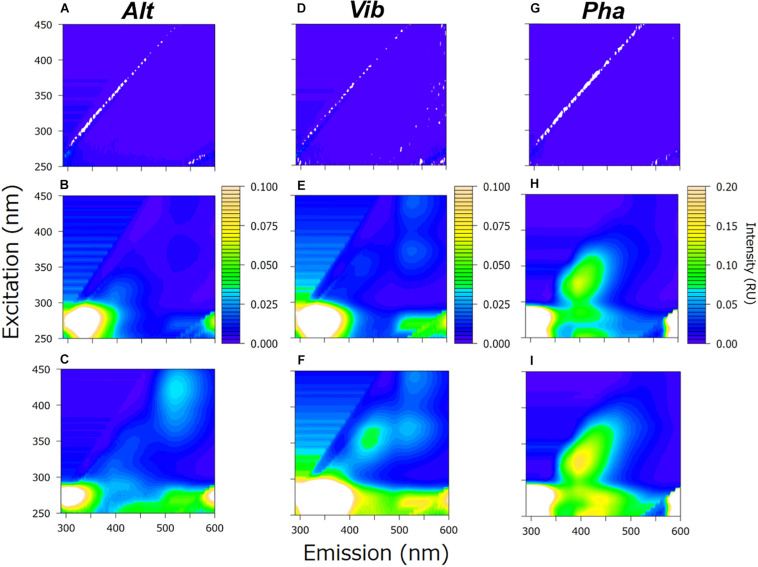
Excitation-emission matrix (EEM) of the experimental treatment at the initiation of the incubation (upper row), the initiation of the stationary phase (middle row) and the end of the incubation (lower row) Alt **(A–C)**, Vib **(D–F)**, and Pha **(G–I)** treatments, respectively. The EEMs obtained from triplicate samples were averaged to produce the figures.

At the end of the incubation periods for all the strains in the experimental treatments, the EEMs of the DOM samples showed distinct fluorescent peaks (Figures 2C,F,I); these fluorescent peaks did not appear at the beginning of the experimental treatments (Figures 2A,D,G) or in the control treatments (Supplementary Figure 1) of three bacterial strains.

The positions of the fluorescent peaks of the FDOM produced by the three bacterial strains are summarized in Table 1. From the positions of the peaks, the protein-like FDOM and humic-like FDOM were grouped. Furthermore, the protein-like FDOM found in the treatment of each strain was separated into tyrosine-like FDOM, which had emission maxima at approximately 300 nm, and tryptophan-like FDOM, which had emission maxima at approximately 330 nm (Mayer et al., 1999; Yamashita and Tanoue, 2003). Two types of protein-like FDOM (AP1: Ex/Em = 275/300 nm and AP2: Ex/Em = 275/330 nm) were found in the Alt treatment, while one type of protein-like FDOM was found in the Vib and Pha treatments. The protein-like FDOM in the Vib treatment was defined as a tryptophan-like FDOM, which had an emission maximum at 340 nm (VP: Ex/Em = 275/340 nm). In the Pha treatment, the protein-like FDOM was defined as a tyrosine-like FDOM (PP: Ex/Em = 280/300 nm).

**TABLE 1:** Peak positions and groups of fluorescent dissolved organic matter produced by three bacterial strains.

Strain	Excitation (nm)	Emission (nm)	Abbreviation	Organic matter group	References
Alt	275	300	AP1	Tyrosine-like group	Mayer et al., 1999
	275	330	AP2	Tryptophan-like group	Mayer et al., 1999
	270	520	AH1	Terrestrial-like group	Stedmon and Markager, 2005
	315	400	AH2	Marine-like group	Coble, 1996
	425	520	AH3	Terrestrial-like group	Stedmon and Markager, 2005
Vib	275	340	VP	Tryptophan-like group	Mayer et al., 1999
	360	450	VH1	Multiple origin group	Yamashita et al., 2010
	370	520	VH2	Terrestrial-like group	Stedmon and Markager, 2005
	440	530	VH3	Terrestrial-like group	Stedmon and Markager, 2005
Pha	280	300	PP	Tyrosine-like group	Mayer et al., 1999
	265	450	PH1	Multiple origin group	Yamashita et al., 2010
	320	390	PH2	Marine -like group	Coble, 1996
	370	450	PH3	Multiple origin group	Yamashita et al., 2010

Humic-like FDOM was also found in the EEMs in all treatments. The humic-like FDOM was further separated into three groups based on comparisons with spectral characteristics of humic-like FDOM reported in previous studies (Coble, 1996, 2007; Stedmon and Markager, 2005; Yamashita et al., 2010; Romera-Castillo et al., 2011; Tanaka et al., 2014; Catalá et al., 2015). Humic-like FDOM, characterized by emission maxima greater than 500 nm, has been generally defined as terrestrial humic-like FDOM in coastal environments (Stedmon and Markager, 2005) and is therefore regarded as a terrestrial-like group in the present study. Two humic-like FDOM peaks in the Alt treatment (AH1: Ex/Em = 270/520 nm, AH3: Ex/Em = 425/520 nm) and the Vib treatment (VH2: Ex/Em = 370/520 nm, VH3: Ex/Em = 440/530 nm) were categorized into the terrestrial-like group.

Humic-like FDOM, which has emission maxima of approximately 400 nm, has been traditionally reported to be derived from marine microbes (Coble, 1996, 2007). Therefore, humic-like FDOM is defined as a marine-like group. One humic-like FDOM found in the Alt treatment (AH2: Ex/Em = 315/400 nm) and the Pha treatment (PH2: Ex/Em = 320/390 nm) was categorized into the marine-like group.

The third humic-like FDOM group, characterized by emission maxima at approximately 450 nm, has been traditionally considered to be of a terrigenous origin (Coble, 1996), although it has been recently reported that humic-like FDOM is also produced by marine microbes (Yamashita et al., 2010; Romera-Castillo et al., 2011; Tanaka et al., 2014; Catalá et al., 2015). Accordingly, humic-like FDOM was classified as a multiple origin group in the present study. One humic-like FDOM in the Vib treatment (VH1: Ex/Em = 360/450 nm) and two humic-like FDOM types in the Pha treatment (PH1: Ex/Em = 265/450 nm, PH3: Ex/Em = 370/450 nm) were categorized into the multiple origin group. It should be noted that all the humic-like FDOM peaks, even in the terrestrial-like group, were produced by the bacterial strains used in the present study.

### Changes Over Time in the Fluorescence Intensity of Individual FDOM Types Produced by Bacterial Strains

**FIGURE 3:**
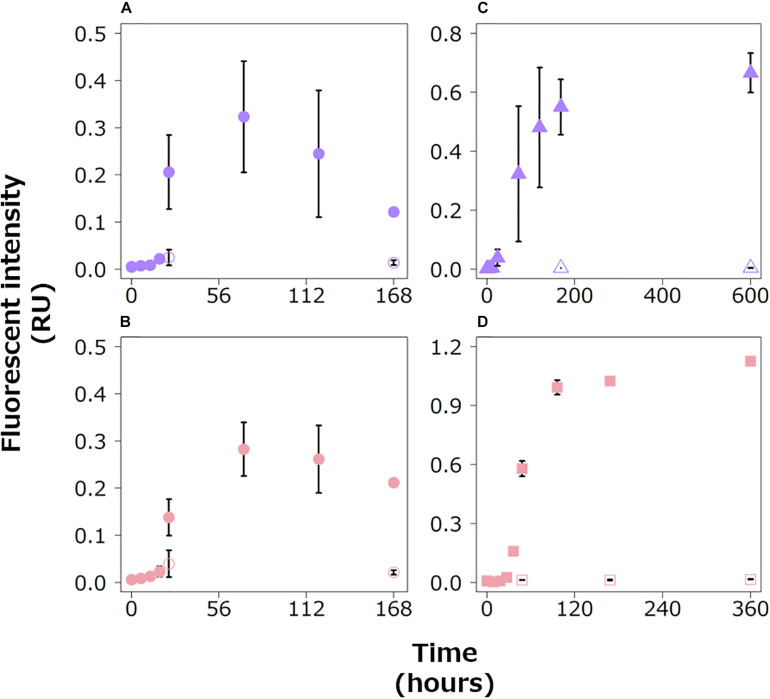
Changes in the fluorescence intensities of protein-like FDOM in the Alt **(A,B)**, Vib **(C)** and Pha treatments **(D)**. The purple and pink symbols indicate tryptophan-like **(A,C)** and tyrosine-like peaks **(B,D)**, respectively. The closed and open symbols represent the experimental treatment and control treatment, respectively. The error bars indicate standard deviation of triplicate incubation bottles.

In the experimental treatments of the three bacterial strains, protein-like FDOM increased during the exponential growth phases, especially in the later stages (Figure 3). Then, the fluorescence intensities of the protein-like FDOM increased during the early part of the stationary phases for the three bacterial strains. Two protein-like FDOM patterns in the Alt treatment tended to decrease from 72 h to the end of the incubation period (Figures 3A,B). The other protein-like FDOM patterns from the Vib and Pha treatments, which were categorized as tryptophan-like and tyrosine-like peaks, respectively, tended to increase throughout the stationary phases. The fluorescence intensities of all the protein-like FDOM types in the control treatment were considerably lower than those in the experimental treatments for the three strains.

**FIGURE 4:**
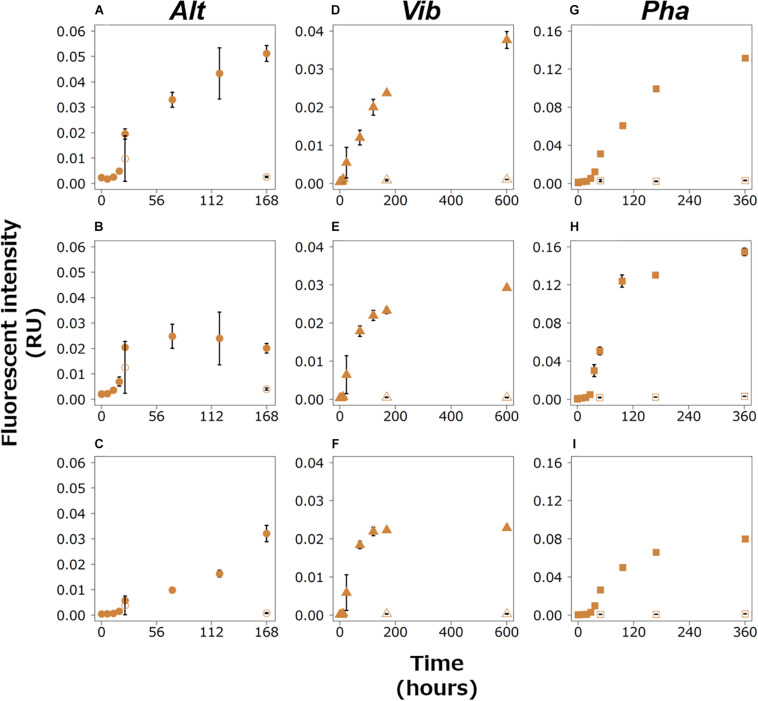
Changes in the fluorescence intensities of peaks categorized as humic-like FDOM in the Alt (AH1, **A**; AH2, **B**; AH3, **C**), Vib (VH1, **D**; VH2, **E**; VH3, **F**), and Pha (PH1, **G**; PH2, **H**; PH3, **I**) treatments. The closed and open symbols represent the experimental treatment and control treatment, respectively. The error bars indicate standard deviation of triplicate incubation bottles.

The fluorescence intensities of all the humic-like FDOM types increased during the exponential growth phases in the experimental treatments of the three bacterial strains (Figure 4). All of them, excluding AH2, continued to increase during the stationary phases. AH2 was relatively stable during the stationary phase of the Alt treatment. The fluorescence intensities of all the humic-like FDOM types in the control treatments were considerably lower than those in the experimental treatments for the three strains.

## Discussion

### Microbial DOC Production From a Labile Substrate

Three marine bacterial strains belonging to different classes or orders were used for a better understanding of the mechanisms of DOM production by the organisms. Glucose was used as the sole carbon source. Previous studies also used glucose as the sole carbon source for bacterial strains or community incubation and indicated that glucose was consumed completely within several days (Ogawa et al., 2001; Kawasaki and Benner, 2006; Wienhausen et al., 2017). Such results of previous studies in combination with increases in bacterial abundances with decreases in the DOC concentrations during the exponential growth phase of the three strains tested (Figure 1) suggest that the bacteria assimilated the glucose and consumed the compound during the exponential growth phase. Therefore, the DOC concentrations detected at the end of the experimental phase were most likely derived from the DOM produced by each bacterial strain.

During the stationary phases, the DOC concentrations were relatively stable for the Alt treatment and slightly increased for the Vib and Pha treatments. The formation of the carbon-rich signature of RDOM with consumption of labile DOM derived from *Spirulina* (a member of cyanobacteria) cells was observed by the 24-h incubation of a microbial community in surface seawater (Hach et al., 2020). Furthermore, the incubation experiment of a bacterial strain, *Alteromonas infernus*, with addition of the labile substrate pyruvate every 48-h showed that release of the labile DOC was not detected during the exponential growth phase (Eichinger et al., 2009). It was assumed that the DOC concentration detected at the end of the exponential growth phase of the three strains were stable due to the absence of labile DOM. Therefore, the changes in DOC concentration indicated that the DOC concentrations at the end of the incubation periods were mainly determined by the direct DOM release during the exponential growth phase with some contribution from the DOM produced during the stationary phase.

**FIGURE 5:**
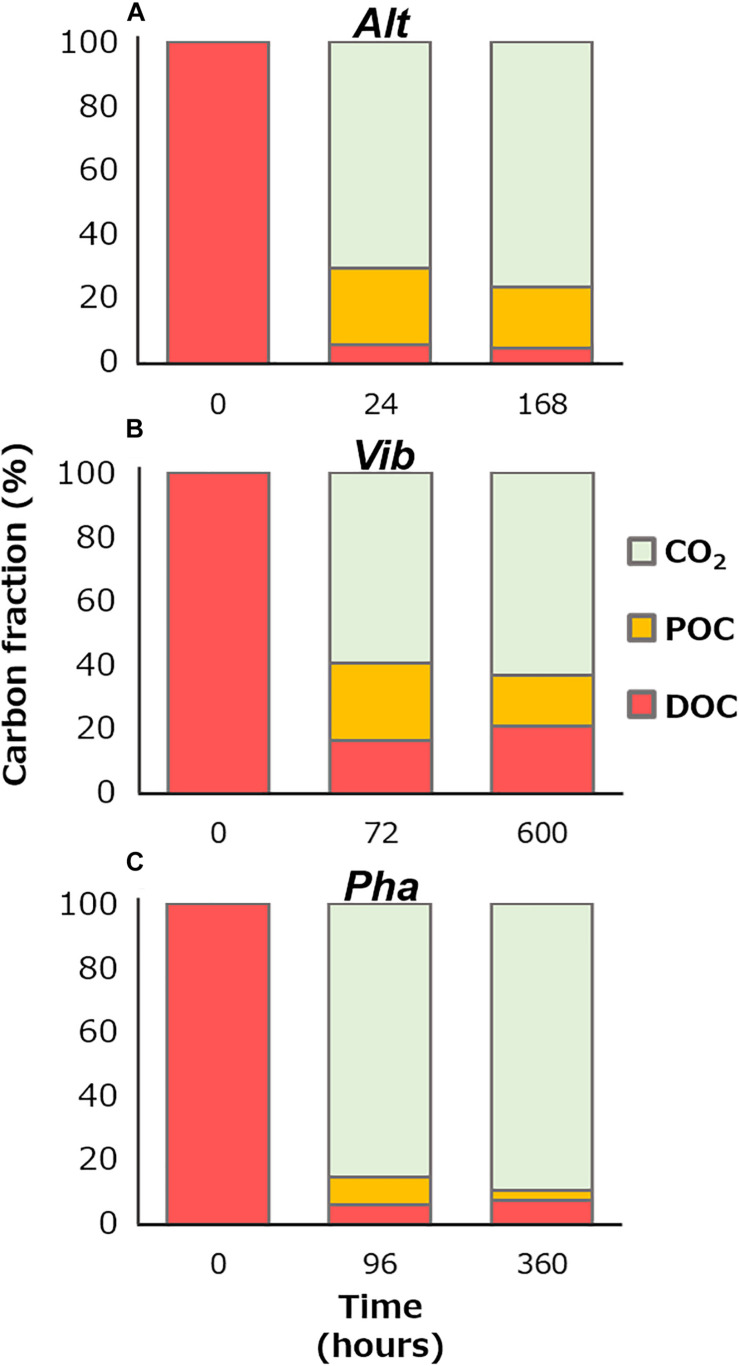
Percentage of carbon species at the initiation of the incubation, the initiation of the stationary phase, and the end of the incubation of the Alt **(A)**, Vib **(B)**, and Pha **(C)**. The amount of remineralization into carbon dioxide was calculated from decreases in TOC concentration from the initiation of the incubation.

Figure 5 shows partition of carbon species, namely CO_2_ by respiration (decrease in TOC), POC, and DOC, at the initiation of the incubation, the initiation of the stationary phases, and the end of the incubation. The bacterial physiological processes for DOM production were thought to differ among the three bacterial strains during the stationary phase. The POC contributed 79, 44, and 28% of the TOC at the end of the incubation periods of the Alt, Vib, and Pha treatments, respectively. The POC at the end of the incubation period included the living bacterial cells and their detritus. The DOC and POC concentrations fluctuated inversely during the stationary phase of the Alt treatment, suggesting that Alt reused the DOC released by themselves to maintain bacterial carbon with the mineralization process.

During the stationary phase of the Vib treatment, the POC concentration and bacterial abundance decreased, but the DOC concentration slightly increased, suggesting that the bacterial cells were degraded (i.e., lysis of the cell body) and released cellular contents into the media. The average decrease in POC concentration during the stationary phase was 85 ± 38 μmol C L^–1^, whereas the increase in DOC concentration during the stationary phase was 46 ± 39 μmol C L^–1^. Therefore, 46 and 54% of the POC was mineralized and transferred to DOC during the lysis of Vib, respectively (Figure 5). Then, the increase in DOC by lysis were estimated of the contributing 21% to the DOC concentration at the end of the incubation period. A previous study, which added a labile substrate to a coastal microbial community, indicated that DOC derived from sloppy feeding of nanoflagellates on bacterial bodies was easily consumed during incubation (Lechtenfeld et al., 2015). The results of previous and present studies indicate that the lysis of bacterial cells is an important process for DOM production and subsequent secondary bacterial production.

The bacterial abundance of the Pha strain during the stationary phase was stable, while the POC concentration decreased. A relationship between bacterial cell volume and carbon content has been observed (Romanova and Sazhin, 2010). Therefore, it is possible that the bacterial cell size decreased during the stationary phase in the Pha treatment. The DOC concentration increased slightly during the stationary phase. A decrease in biomass with DOM release was also found during incubation with a bacterial strain of *A. infernus* (Eichinger et al., 2009). The average decrease in POC concentration during the stationary phase of the Pha treatment was 55 ± 7 μmol C L^–1^, and the average increase in DOC concentration was 13 ± 8 μmol C L^–1^. Therefore, the decrease in POC concentration during the stationary phase resulted in 77% of the remineralization and 23% of the releases as DOC during the consumption of cellular materials (Figure 5). The DOC released during the consumption of cellular materials was estimated of contributing to 17% of the DOC concentration at the end of the incubation of the Pha treatment.

A major part of the remnant DOC at the end of the incubation period was considered to be generally unavailable for each strain. The concentrations of bacterial DOC could be calculated by subtracting the DOC concentration at the end of the control treatment from that at the end of the experimental treatments during the incubation of each strain. The efficiency of bacterial DOC production (%) from labile substrates was calculated based on the ratio of the concentration of bacterial DOC at the end of incubation to that at the beginning of incubation (Figure 5). The efficiencies of bacterial DOC production are summarized in Table 2. The efficiency of Vib (20%) was the highest among the three strains and was approximately four times higher than those of Alt and Pha (4 and 6%, respectively). The efficiencies of Alt and Pha were similar to the efficiencies found by previous studies (3–7%) of microbial communities (Ogawa et al., 2001; Shimotori et al., 2009; Koch et al., 2014; Lechtenfeld et al., 2015) and isolated strains (Gruber et al., 2006). The experimental evidence obtained from *in vitro* microbial incubation (Ogawa et al., 2001; Gruber et al., 2006; Shimotori et al., 2009; Koch et al., 2014; Lechtenfeld et al., 2015), including this study, indicates that Vib is likely a key species for the efficient production of bacterially derived DOC; thus, differences in bacterial community composition affect the efficiency of bacterial DOC production from simple labile substrates.

**TABLE 2:** Efficiencies of dissolved organic carbon (DOC) production from glucose by the bacterial strains.

Strain	Incubation time (hours)	DOC at the end of incubations (μmol C L^–1^)	Efficiency of DOC production (%)
Alt	168	51 ± 4	4.2 ± 0.5
Vib	600	218 ± 8	19.8 ± 0.8
Pha	360	75 ± 43	5.7 ± 0.9

### Bacterial FDOM Production With the Consumption of a Labile Substrate During the Exponential Growth Phase

The results of the EEMs for the experimental treatments of all the strains (Figure 2) indicated that FDOM was produced by the three bacterial strains during the incubations and was not due to contamination during incubation or of the background medium. The three bacterial strains produced FDOM, which contained protein-like and humic-like substances, during the exponential growth phase, suggesting that the bacteria actively released FDOM during the growth phase, in which they consumed the labile substrate. The fluorescence intensities of the humic-like FDOM did not decrease during the exponential growth phase or the stationary phase (as discussed below). Furthermore, the bacterial-derived humic-like FDOM has been shown to be recalcitrant on the time scale of deep ocean circulation (Yamashita and Tanoue, 2008; Catalá et al., 2015). These results suggested that the heterotrophic bacteria produced recalcitrant humic-like FDOM using simple substrates, such as glucose, during their active growth. The production of recalcitrant humic-like FDOM during the exponential growth phase has also been reported from previous incubation experiments with natural microbial communities (Kramer and Herndl, 2004; Lønborg et al., 2009; Shimotori et al., 2009; Arai et al., 2018). The results of these previous studies as well as this study suggested that broad heterotrophic bacterial species can actively produce humic-like FDOM during the exponential growth phase. However, it should be noted that some previous studies suggested that a part of humic-like FDOM produced by microbial community is bio-degradable (Shimotori et al., 2009; Arai et al., 2018).

The efficiency of humic-like FDOM production with the consumption of a labile substrate was compared among the three bacterial strains. A regression analysis between DOC concentrations and fluorescence intensities of the humic-like FDOM was performed during the exponential growth phase of each strain (Table 3 and Supplementary Figure 2). Significant negative linear relationships were found between all the humic-like FDOM types and DOC concentrations, suggesting that the relationship between the production rate of humic-like FDOM and the consumption of glucose was relatively constant during the exponential growth phase. The slope of the regression indicates the efficiency of humic-like FDOM production with the consumption of a labile substrate. Interestingly, the slopes were not largely different among the humic-like FDOM produced by each strain (Table 3). On the other hand, the slopes seemed to be different among the three bacterial strains, namely, the slope was largest in the Pha treatment but was smallest in the Vib treatment (Table 3). Such a difference in the efficiency of humic-like FDOM production during the exponential growth phase suggested that although a variety of bacteria can produce humic-like FDOM, particular bacterial species predominantly produce humic-like FDOM when labile DOM is supplied.

**TABLE 3:** The results of a regression analyses between humic-like FDOM and other parameters in the treatments of three bacterial strain.

Strain	*X*	*Y*	Exponential growth phase			Stationary phase		
			*p*-value	*R*^2^	Slope (×10^–4^)	*p*-value	*R*^2^	Slope (×10^–4^)
Alt	DOC	AH1	**	0.77	−0.176		0.04	−1.12
		AH2	**	0.69	−0.270		0.06	−0.588
		AH3	**	0.84	−0.0614		0.96	−1.42
	TOC	AH1	**	0.93	−0.207	**	0.84	−5.21
		AH2	**	0.86	−0.313		0.007	−0.189
		AH3	**	0.90	−0.0657	**	0.77	−3.96
	POC	AH1	–	–	–		0.29	−2.27
		AH2	–	–	–		0.02	0.211
		AH3	–	–	–		0.18	−1.43
	AP1	AH1	**	0.70	1400		0.32	1000
		AH2	**	0.84	2500	**	0.55	560
		AH3	**	0.64	450		0.02	230
	AP2	AH1	**	0.63	1300		0.18	−400
		AH2	*	0.56	2100	**	0.65	390
		AH3	**	0.80	510		0.001	−40
Vib	DOC	VH1	**	0.76	−0.131	**	0.40	2.08
		VH2	**	0.77	−0.184	**	0.46	0.991
		VH3	**	0.66	−0.122	**	0.60	0.507
	TOC	VH1		0.38	−0.0338		0.29	−2.56
		VH2		0.37	−0.0322		0.28	−1.13
		VH3		0.02	−0.0092		0.34	−0.547
	POC	VH1	*–*	–	–	**	0.62	−1.82
		VH2	–	–	–	**	0.65	−0.840
		VH3	–	–	–	**	0.86	−0.423
	VP	VH1	**	0.81	1000	**	0.55	380
		VH2	**	0.80	1000	**	0.59	180
		VH3	**	0.60	1000	**	0.63	81
Pha	DOC	PH1	**	0.99	−0.378	*	0.56	66.3
		PH2	**	0.96	−0.673	*	0.51	14.3
		PH3	**	0.98	−0.333	*	0.60	13.9
	TOC	PH1	**	0.97	−0.434	**	0.76	−12.7
		PH2	**	0.96	−0.779		0.41	−4.39
		PH3	**	0.97	−0.383	**	0.73	−5.29
	POC	PH1	–	–	–	**	0.88	−11.3
		PH2	–	–	–	*	0.54	−4.17
		PH3	–	–	–	**	0.86	−4.76
	PP	PH1	**	0.99	500	**	0.79	4300
		PH2	**	0.89	860	**	0.83	2100
		PH3	**	0.99	440	**	0.80	1800

*p < 0.05, **p < 0.01, Abbreviations are the same as those in Table 1.

### Bacterial Production of FDOM During the Stationary Phase

The fluorescence intensities of humic-like FDOM continued to increase during the stationary phase, except for one humic-like FDOM produced by Alt (AH2). Such changes in humic-like FDOM during the incubations suggest that recalcitrant humic-like FDOM produced by the three bacterial strains accumulated during the incubations. It should be noted that the patterns of change in the humic-like FDOM during the stationary phase were different among the three bacterial strains, possibly due to the different production mechanisms of recalcitrant humic-like FDOM accompanied by the physiology of each strain. Each production mechanism was evaluated based on the linear regression analysis between humic-like FDOM and the DOC concentration (Supplementary Figure 3), TOC concentration (Supplementary Figure 5), and POC concentration (Supplementary Figure 6) during the stationary phase (Table 3).

In the Alt treatment, the humic-like FDOM in the terrestrial-like group (AH1 and AH3) increased, while the protein-like FDOM tended to decrease during the stationary phase (Figures 3A,B, 4A,C), suggesting that humic-like FDOM was produced with the recycling of ambient DOM derived from Alt. The fluorescence intensities of AH1 and AH3 were not significantly related to DOC concentrations in the Alt treatment (Figures 1B, 4A,C). In contrast, significant negative linear relations were evident between the TOC concentration and fluorescence intensities of AH1 and AH3, suggesting that Alt produced humic-like FDOM in the terrestrial-like group with the mineralization of organic matter (Table 3 and Supplementary Figure 5). The other humic-like FDOM produced by Alt, namely, AH2 belonging to the marine-like group, which has been considered to be recalcitrant in the ocean (Yamashita and Tanoue, 2008; Catalá et al., 2015), was not produced during the stationary phase.

In the Vib experiment, all the humic-like FDOM types (i.e., VH1, VH2, and VH3) increased with decreases in bacterial abundance and POC concentration during the stationary phase, including a part of the death phase (Figures 1E,G, 4D–F), suggesting that humic-like FDOM was produced with decreasing biomass. In incubation experiments without predators, the decrease in bacterial biomass should be caused by two mechanisms, namely, the lysis of bacteria (Riemann and Middelboe, 2002) and reserve consumption in bacterial bodies (Eichinger et al., 2009). The decrease in the biomass of Vib during the stationary phase (including a part of the death phase) was likely caused by bacterial lysis due to a simultaneous decrease in bacterial abundance. The humic-like FDOM was negatively related to POC concentration but not to TOC concentration during the stationary phase (Table 3 and Supplementary Figures 5, 6). Such relationships suggested that humic-like FDOM was not produced in association with bacterial mineralization, including the consumption of the reserve in biomass, but was released from the bacteria through lysis. It is interesting to note that all the FDOM types, including the protein-like FDOM types, increased throughout the incubation period of the Vib treatment. Positive linear relationships were evident between the humic-like FDOM and the DOC concentration as well as the protein-like FDOM (Table 3 and Supplementary Figures 3, 8), suggesting that not only humic-like FDOM but also DOM derived from the lysis of Vib accumulated throughout the incubation periods.

The humic-like FDOM in the Pha treatment increased with a decrease in the POC concentration during the stationary phase, although the bacterial abundance did not decrease (Figures 1I,K, 4G–I). Two humic-like FDOM types (PH1 and PH3) in the multiple origin group were negatively related to TOC as well as POC concentrations, possibly equivalent to the bacterial biomass, during the stationary phase (Table 3 and Supplementary Figures 5, 6), suggesting that a part of the humic-like FDOM was produced by the consumption of cellular materials. A decrease in biomass with DOM release was found during the incubation of the bacterial strain *A. infernus* (Eichinger et al., 2009). The DOC concentration was positively related to recalcitrant humic-like FDOM during the stationary phase (Table 3 and Supplementary Figure 3), suggesting that DOM produced by Pha during the stationary phase was possibly recalcitrant and accumulated throughout the incubation.

It is interesting to note that the composition of the bacterial FDOM (the slope of the linear regression between the protein-like FDOM and the humic-like FDOM) during the stationary phases was different between Pha and Vib (Table 3 and Supplementary Figures 8, 10). Such differences in the composition were likely caused by different mechanisms of DOM release; that is, more humic-rich DOM was produced during the consumption of cellular materials (in the case of Pha) than as a result of cell lysis (in the case of Vib).

### The Differences in Bacterial FDOM Composition Between the Exponential Growth and Stationary Phases

All three types of humic-like FDOM were produced by Alt during the exponential growth phase, but only the two were produced during the stationary phase. These results indicate that the composition of microbially derived humic-like FDOM was different between the growth phases, namely, the physiological states, which could be partially controlled by differences in substrate, such as glucose or ambient DOM derived from Alt.

The levels of humic-like FDOM were negatively related to the TOC concentration during the exponential growth and stationary phases of the Alt treatment, except for AH2 in the stationary phase (Table 3 and Supplementary Figures 4, 5). All three humic-like FDOM types and the two humic-like FDOM types in the multiple origin group were also negatively related to the TOC concentration during the exponential growth and stationary phases of the Pha treatment, respectively. However, the levels of the humic-like FDOM types were not related to the TOC concentration during the exponential growth and stationary phases of the Vib treatment. The slopes of the regressions between the TOC concentration and fluorescence intensity of the humic-like FDOM during the stationary phases were larger than those during the exponential growth phases of both the Alt and Pha treatments, suggesting that starvation in a labile substrate possibly induced a higher production rate of humic-like FDOM and *vice versa*.

The humic-like and protein-like FDOM continuously increased throughout the incubations of the Vib and Pha treatments, even though protein-like FDOM was reused during the stationary phase of the Alt treatment (Figure 3; Goto et al., 2017). All the humic-like FDOM types were positively related to the protein-like FDOM types during the exponential growth and stationary phases of the Vib and Pha treatments (Table 3 and Supplementary Figures 7–10). The slopes of the positive linear relationships between the two parameters during the exponential growth phase showed higher values than those during the stationary phase of the Vib treatment (Table 3 and Supplementary Figures 7, 8). The slope, namely, the ratio of humic-like FDOM to protein-like FDOM, was probably controlled by mechanisms of DOM release. That is, protein-like FDOM-poor DOM was released with active extracellular excretion during the exponential growth phase, while protein-like FDOM-rich DOM was passively released by the lysis of bacteria during the stationary phase, including a part of the death phase. A previous study reported that viral lysis of bacteria induced the release of proteins (Riemann and Middelboe, 2002). In contrast, Zhao et al. (2017) pointed out that cyanobacteria released humic-like FDOM due to viral lysis rather than active excretion during exponential growth. Such experimental evidence suggests that viral lysis is an important process for releasing protein-like and humic-like FDOM and therefore that bacterial physiology regulates the composition of DOM released to ambient seawater.

The slopes of the positive linear relationships between the protein-like FDOM and humic-like FDOM were smaller during the exponential growth phase than during the stationary phase in the Pha treatment (Table 3 and Supplementary Figures 9, 10), indicating that the FDOM produced by Pha during the stationary phase showed a higher relative abundance of humic-like FDOM than that during the exponential growth phase. Such differences suggested that Pha excreted protein-rich DOM during the consumption of the labile substrate but produced protein-poor DOM during the consumption of cellular materials.

## Conclusion

The efficiencies of DOM production from simple substrates were different among the three bacterial strains (i.e., Alt, 4%; Vib, 20%; and Pha, 6%). Interestingly, Vib produced DOM efficiently compared with the other strains. The bacterial DOC detected at the end of the incubations was recalcitrant for each bacterial strain. However, the degradability of the bacterial DOC for the other bacterial species is unknown. The incubation experiments of the bacterial DOC with other bacterial species are apparently necessary for a better understanding of RDOM production by marine bacteria. Humic-like FDOM, which can be considered RDOM, was produced by the three bacterial strains. The present study found, for the first time, that relative to the consumption of labile substrate during the exponential growth phase, the efficiency of humic-like FDOM production differed among the bacterial species, suggesting that the existence of key species with the ability to efficiently produce RDOM in the ocean.

In addition, four mechanisms of humic-like FDOM production were suggested based on the incubation of the distinct strains. (1) Active excretion with the consumption of labile substrate during the exponential growth phases. (2) Production with the consumption of extracellular compounds released by Alt. (3) Release through the lysis of Vib. (4) Production with the consumption of cellular materials by Pha. Two production mechanisms of humic-like FDOM, i.e., (2) and (4), showed higher efficiency against the remineralization of organic matter during the stationary phase than during the exponential growth phase. These data indicate that the efficiency of humic-like FDOM production depends on bacterial species as well as bacterial physiology and thus suggest that changes in the species composition and/or physiology of the microbial community are key factors controlling RDOM production in the ocean.

## Data Availability Statement

The datasets generated for this study are available on request to the corresponding author.

## Author Contributions

All the authors contributed to the design of the study. SG performed the incubation experiment, sample measurements, and data analyses with the help of YT, KS, and YY. SG wrote the initial draft of the manuscript. All authors contributed to its revision.

## Conflict of Interest

The authors declare that the research was conducted in the absence of any commercial or financial relationships that could be construed as a potential conflict of interest.
