# Supplementary material for: Evaluation of the Production of Dissolved Organic Matter by Three Marine Bacterial Strains
Source: Front Microbiol. 2020 Oct 15;11:584419. doi: 10.3389/fmicb.2020.584419 (PMC7593260; doi:10.3389/fmicb.2020.584419)
Supplement: Supplementary file 1 [file Presentation_1.PPTX]

## Slide 1
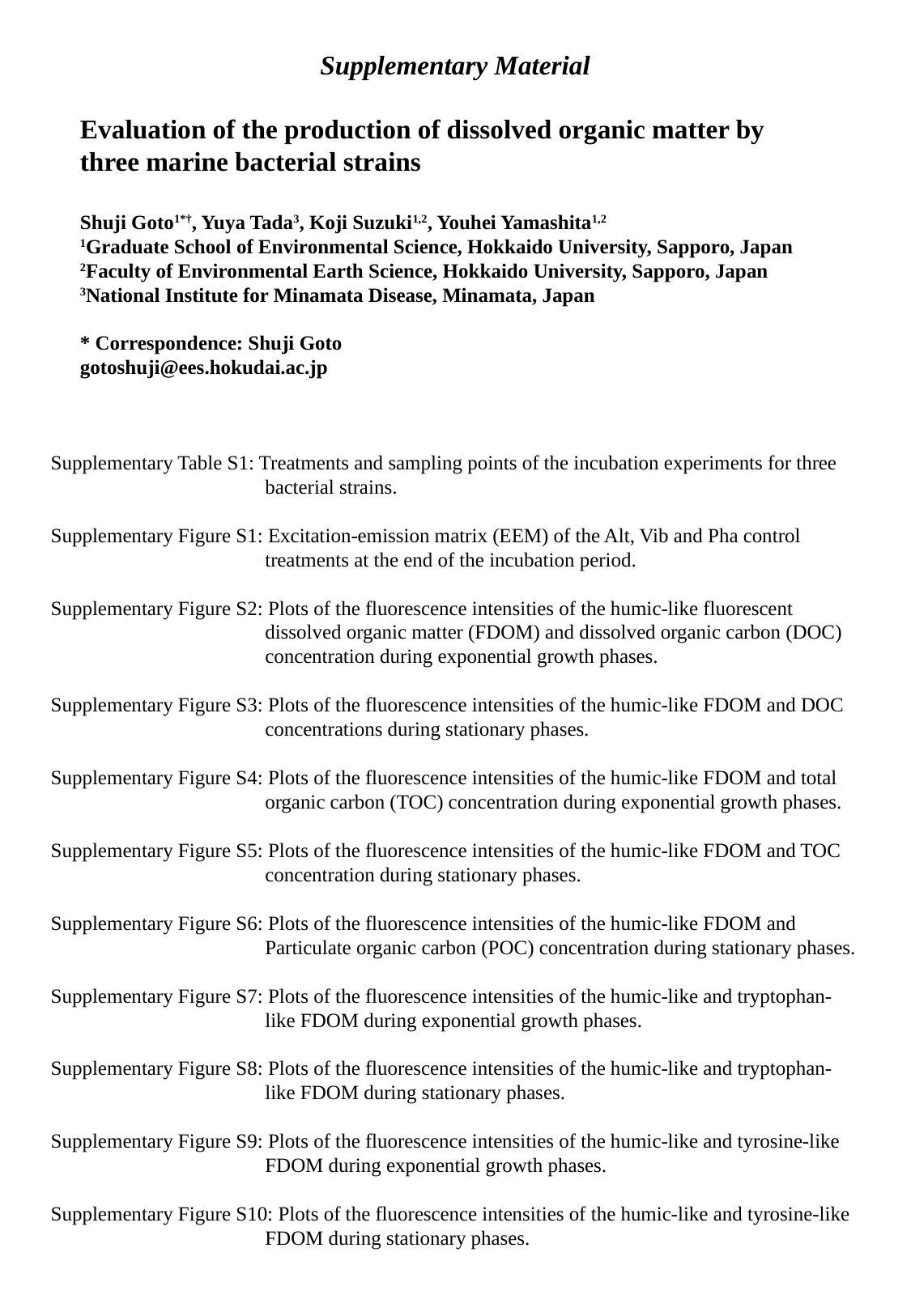

Supplementary Material
Evaluation of the production of dissolved organic matter by three marine bacterial strains
Shuji Goto1*†, Yuya Tada3, Koji Suzuki1,2, Youhei Yamashita1,2
1Graduate School of Environmental Science, Hokkaido University, Sapporo, Japan
2Faculty of Environmental Earth Science, Hokkaido University, Sapporo, Japan
3National Institute for Minamata Disease, Minamata, Japan
* Correspondence: Shuji Goto
gotoshuji@ees.hokudai.ac.jp
Supplementary Table S1: Treatments and sampling points of the incubation experiments for three bacterial strains.
Supplementary Figure S1: Excitation-emission matrix (EEM) of the Alt, Vib and Pha control treatments at the end of the incubation period.
Supplementary Figure S2: Plots of the fluorescence intensities of the humic-like fluorescent dissolved organic matter (FDOM) and dissolved organic carbon (DOC) concentration during exponential growth phases.
Supplementary Figure S3: Plots of the fluorescence intensities of the humic-like FDOM and DOC concentrations during stationary phases.
Supplementary Figure S4: Plots of the fluorescence intensities of the humic-like FDOM and total organic carbon (TOC) concentration during exponential growth phases.
Supplementary Figure S5: Plots of the fluorescence intensities of the humic-like FDOM and TOC concentration during stationary phases.
Supplementary Figure S6: Plots of the fluorescence intensities of the humic-like FDOM and Particulate organic carbon (POC) concentration during stationary phases.
Supplementary Figure S7: Plots of the fluorescence intensities of the humic-like and tryptophan-like FDOM during exponential growth phases.
Supplementary Figure S8: Plots of the fluorescence intensities of the humic-like and tryptophan-like FDOM during stationary phases.
Supplementary Figure S9: Plots of the fluorescence intensities of the humic-like and tyrosine-like FDOM during exponential growth phases.
Supplementary Figure S10: Plots of the fluorescence intensities of the humic-like and tyrosine-like FDOM during stationary phases.

## Slide 2
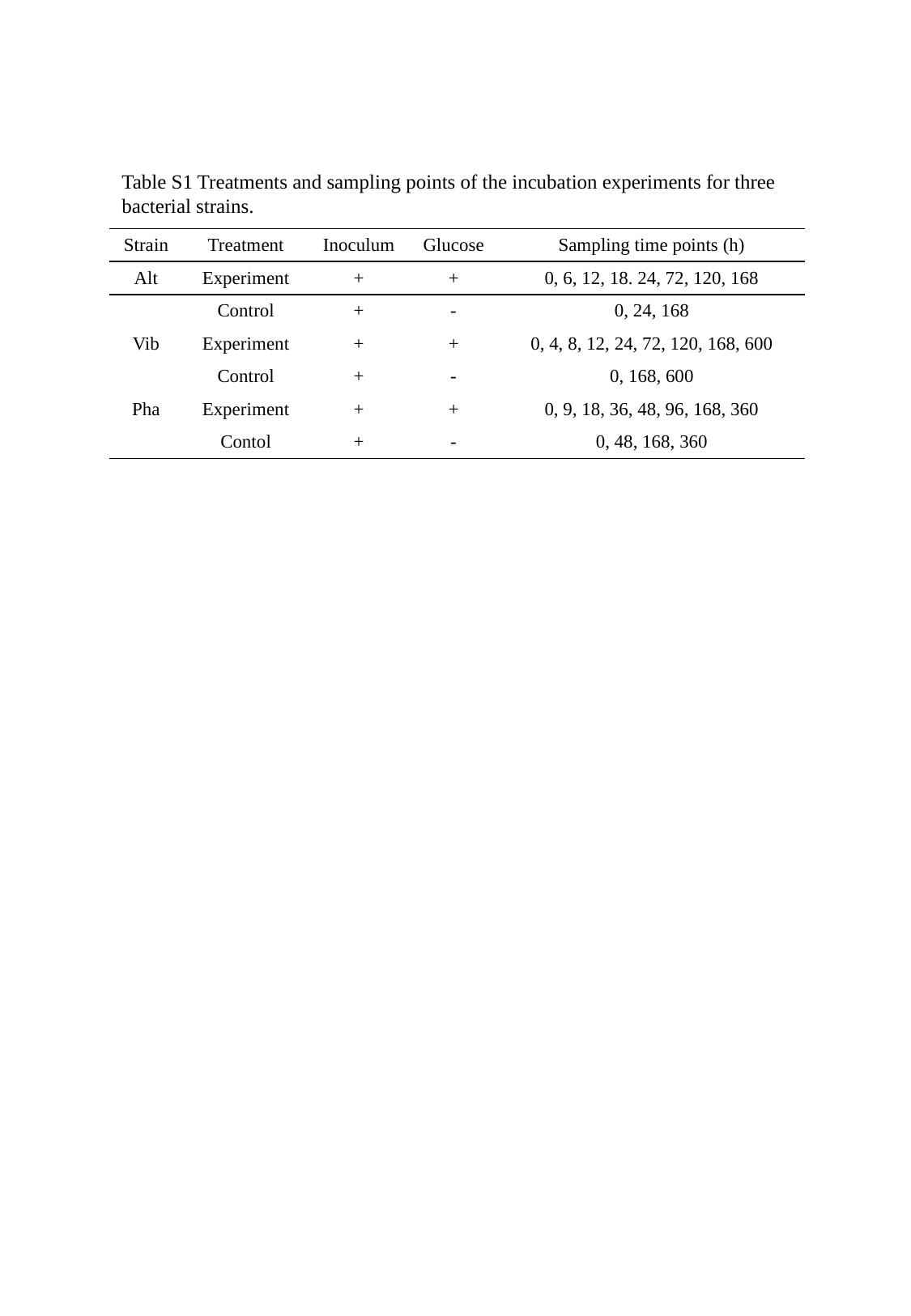

Table S1 Treatments and sampling points of the incubation experiments for three bacterial strains.
| Strain | Treatment | Inoculum | Glucose | Sampling time points (h) |
| --- | --- | --- | --- | --- |
| Alt | Experiment | + | + | 0, 6, 12, 18. 24, 72, 120, 168 |
| | Control | + | - | 0, 24, 168 |
| Vib | Experiment | + | + | 0, 4, 8, 12, 24, 72, 120, 168, 600 |
| | Control | + | - | 0, 168, 600 |
| Pha | Experiment | + | + | 0, 9, 18, 36, 48, 96, 168, 360 |
| | Contol | + | - | 0, 48, 168, 360 |
| | | | | |

## Slide 3
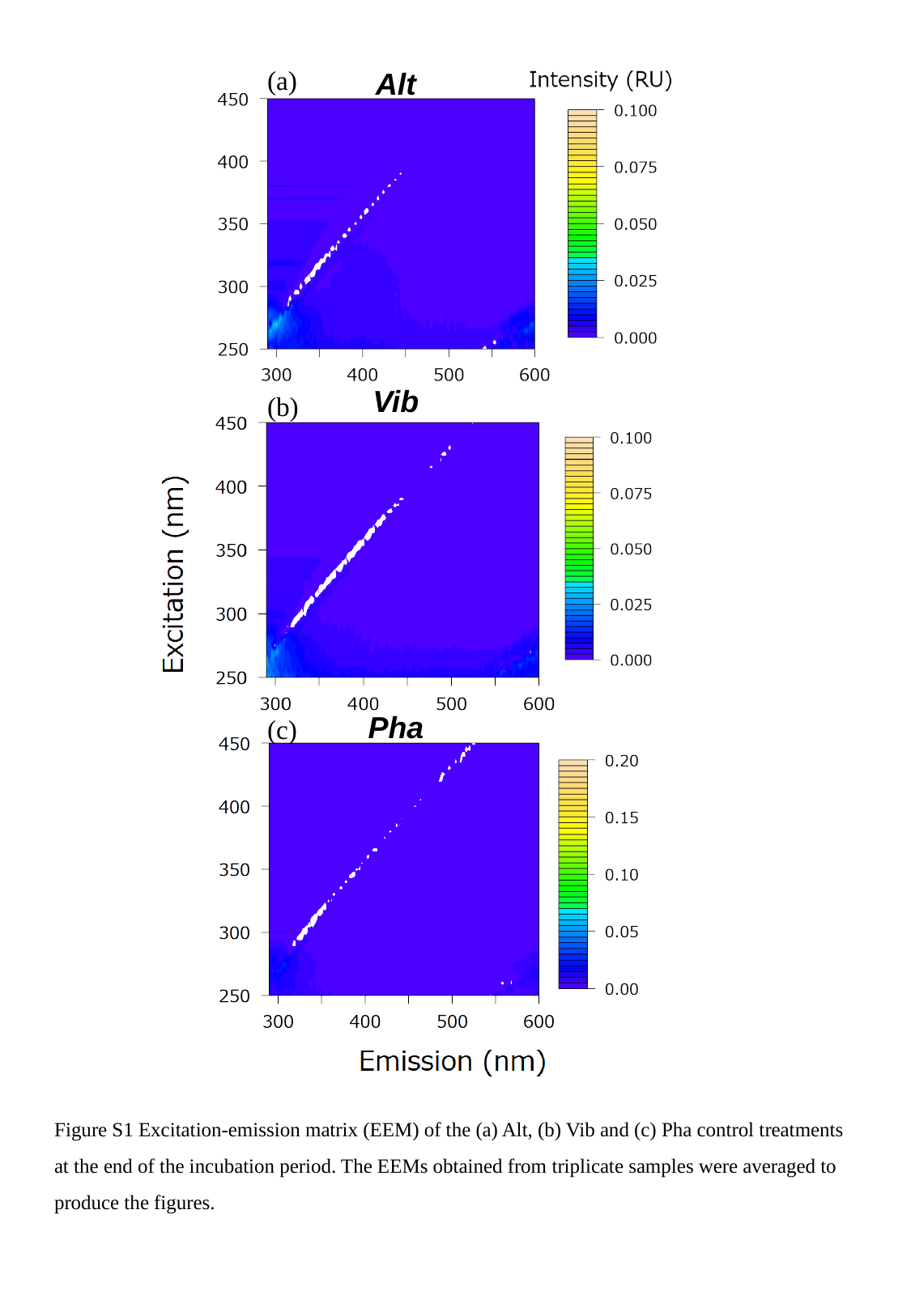

(a)
Alt
Vib
(b)
Pha
(c)
Figure S1 Excitation-emission matrix (EEM) of the (a) Alt, (b) Vib and (c) Pha control treatments at the end of the incubation period. The EEMs obtained from triplicate samples were averaged to produce the figures.

## Slide 4
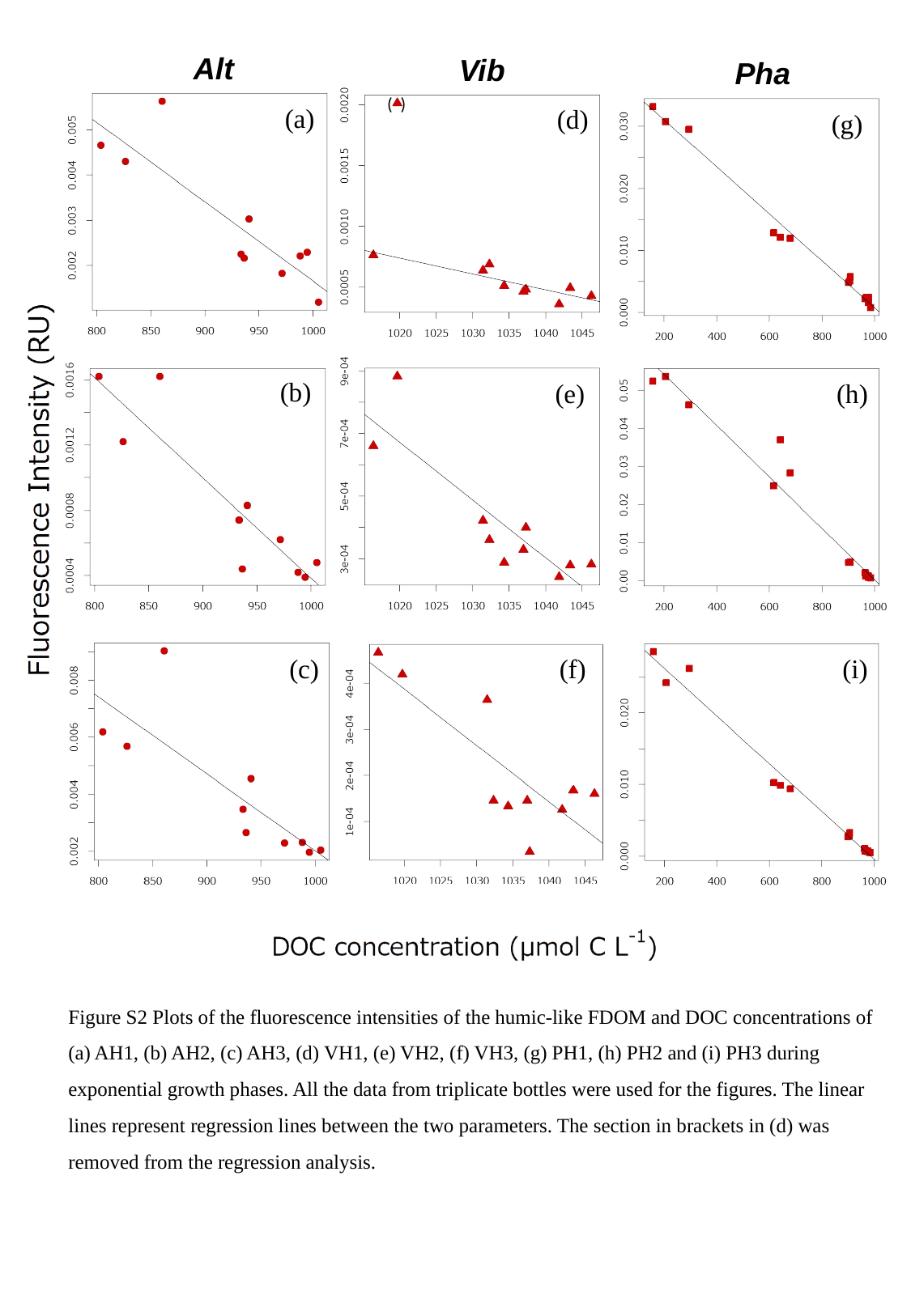

Alt
Vib
Pha
( )
(a)
(d)
(g)
(b)
(h)
(e)
(i)
(f)
(c)
Figure S2 Plots of the fluorescence intensities of the humic-like FDOM and DOC concentrations of (a) AH1, (b) AH2, (c) AH3, (d) VH1, (e) VH2, (f) VH3, (g) PH1, (h) PH2 and (i) PH3 during exponential growth phases. All the data from triplicate bottles were used for the figures. The linear lines represent regression lines between the two parameters. The section in brackets in (d) was removed from the regression analysis.

## Slide 5
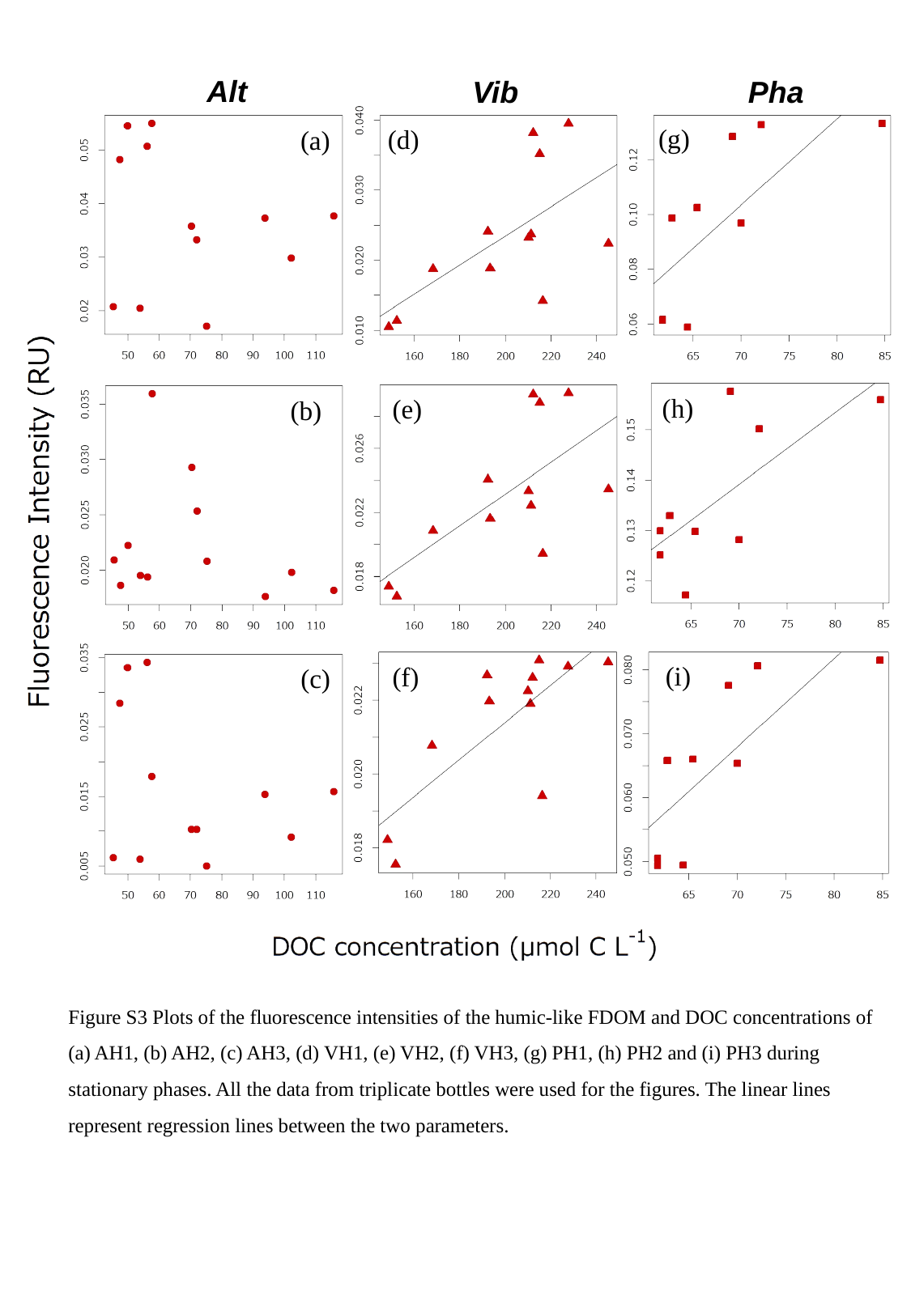

Alt
Vib
Pha
(g)
(d)
(a)
(h)
(e)
(b)
(i)
(f)
(c)
Figure S3 Plots of the fluorescence intensities of the humic-like FDOM and DOC concentrations of (a) AH1, (b) AH2, (c) AH3, (d) VH1, (e) VH2, (f) VH3, (g) PH1, (h) PH2 and (i) PH3 during stationary phases. All the data from triplicate bottles were used for the figures. The linear lines represent regression lines between the two parameters.

## Slide 6
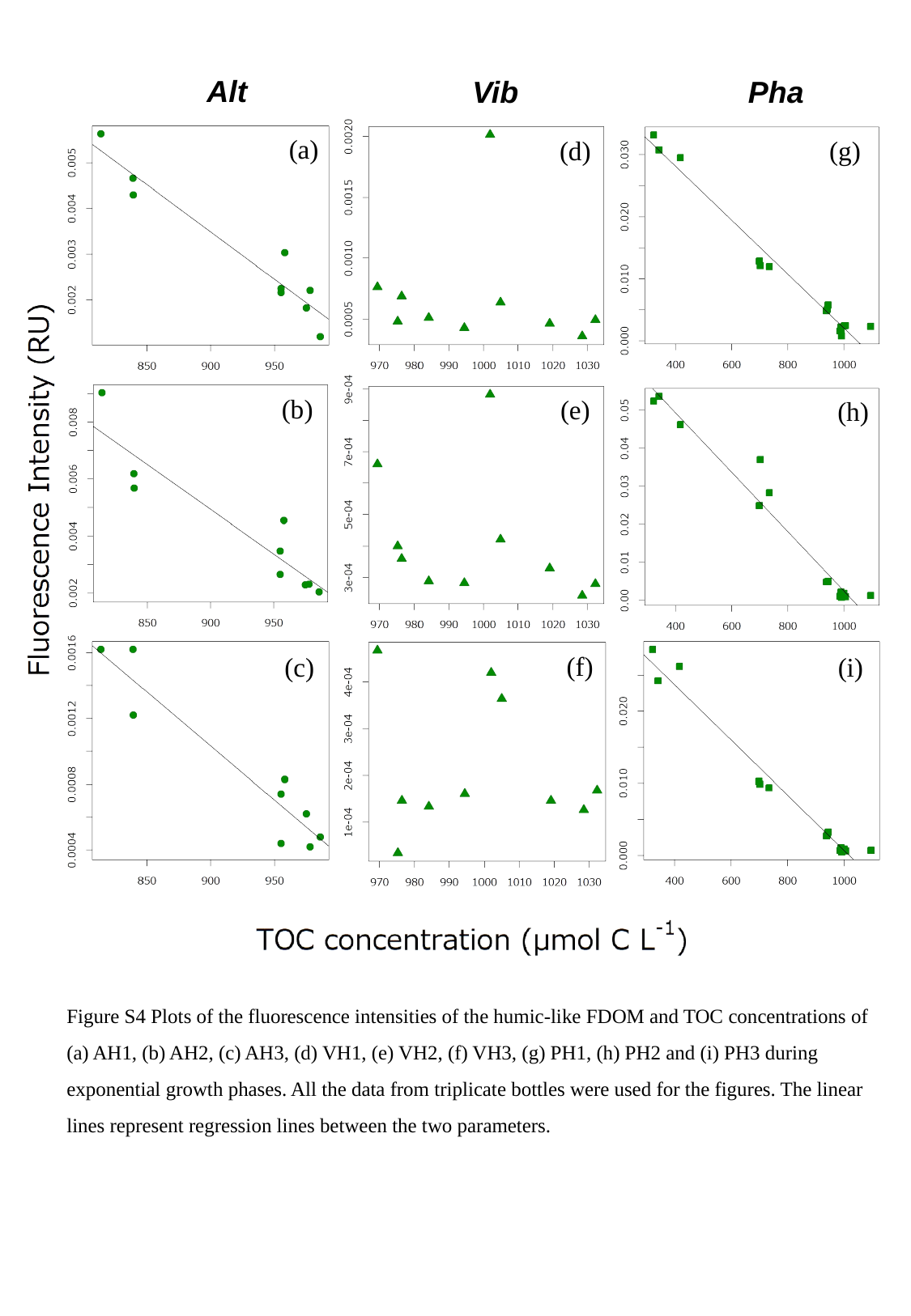

Alt
Vib
Pha
(a)
(g)
(d)
(b)
(e)
(h)
(f)
(c)
(i)
Figure S4 Plots of the fluorescence intensities of the humic-like FDOM and TOC concentrations of (a) AH1, (b) AH2, (c) AH3, (d) VH1, (e) VH2, (f) VH3, (g) PH1, (h) PH2 and (i) PH3 during exponential growth phases. All the data from triplicate bottles were used for the figures. The linear lines represent regression lines between the two parameters.

## Slide 7
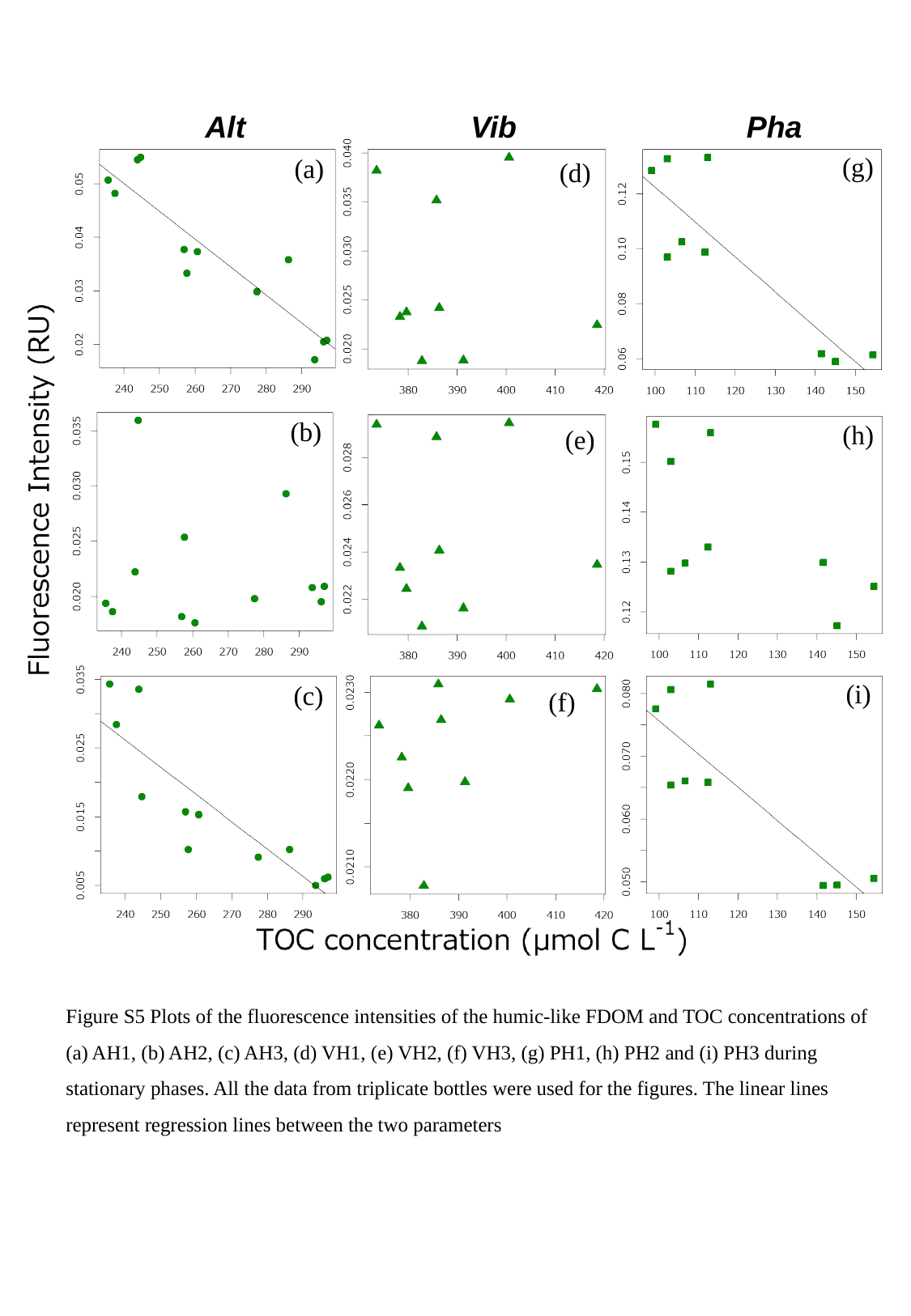

Alt
Vib
Pha
(g)
(a)
(d)
(b)
(h)
(e)
(i)
(c)
(f)
Figure S5 Plots of the fluorescence intensities of the humic-like FDOM and TOC concentrations of (a) AH1, (b) AH2, (c) AH3, (d) VH1, (e) VH2, (f) VH3, (g) PH1, (h) PH2 and (i) PH3 during stationary phases. All the data from triplicate bottles were used for the figures. The linear lines represent regression lines between the two parameters

## Slide 8
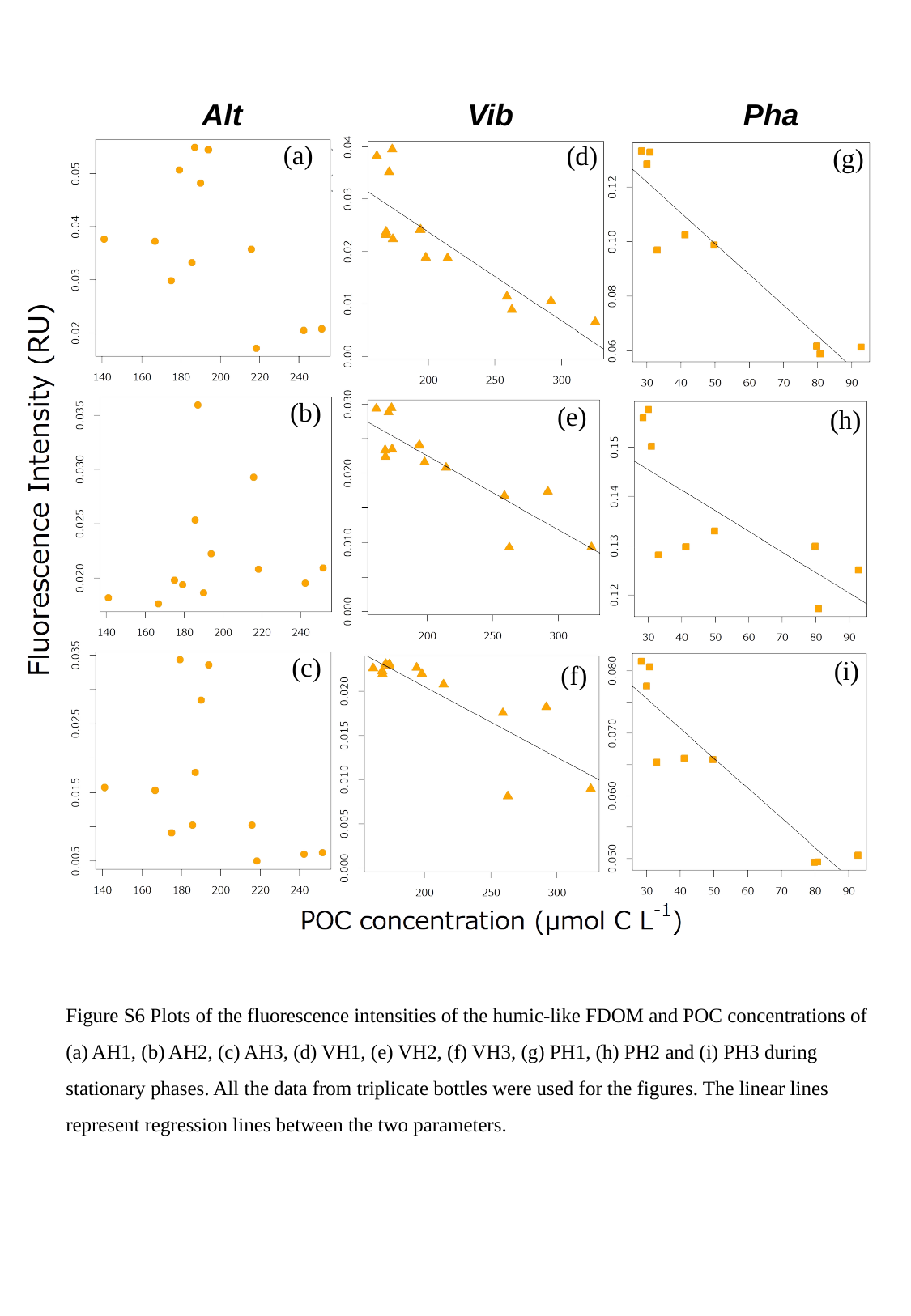

Alt
Vib
Pha
(a)
(d)
(g)
(b)
(e)
(h)
(c)
(i)
(f)
Figure S6 Plots of the fluorescence intensities of the humic-like FDOM and POC concentrations of (a) AH1, (b) AH2, (c) AH3, (d) VH1, (e) VH2, (f) VH3, (g) PH1, (h) PH2 and (i) PH3 during stationary phases. All the data from triplicate bottles were used for the figures. The linear lines represent regression lines between the two parameters.

## Slide 9
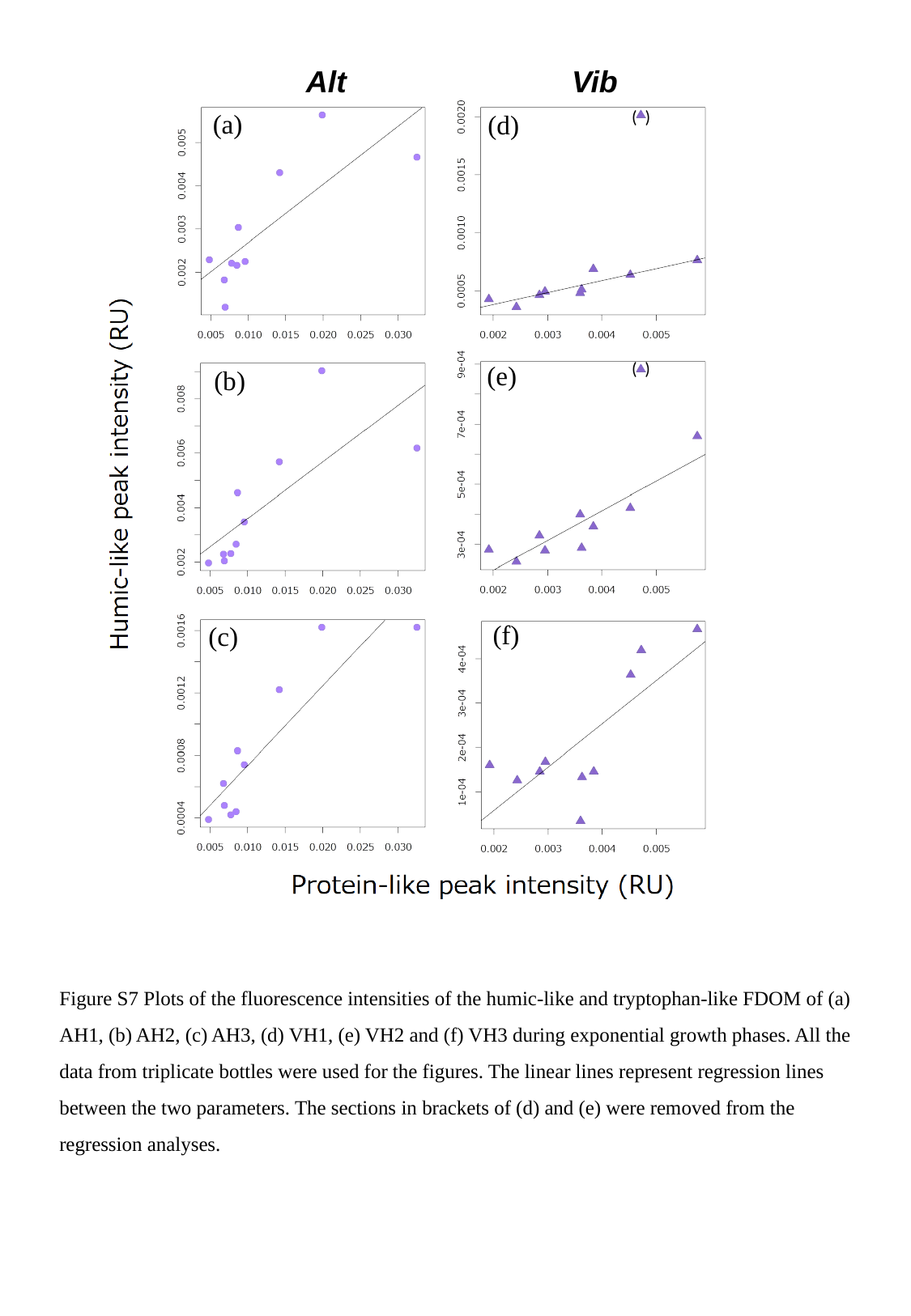

Alt
Vib
( )
(a)
(d)
( )
(e)
(b)
(f)
(c)
Figure S7 Plots of the fluorescence intensities of the humic-like and tryptophan-like FDOM of (a) AH1, (b) AH2, (c) AH3, (d) VH1, (e) VH2 and (f) VH3 during exponential growth phases. All the data from triplicate bottles were used for the figures. The linear lines represent regression lines between the two parameters. The sections in brackets of (d) and (e) were removed from the regression analyses.

## Slide 10
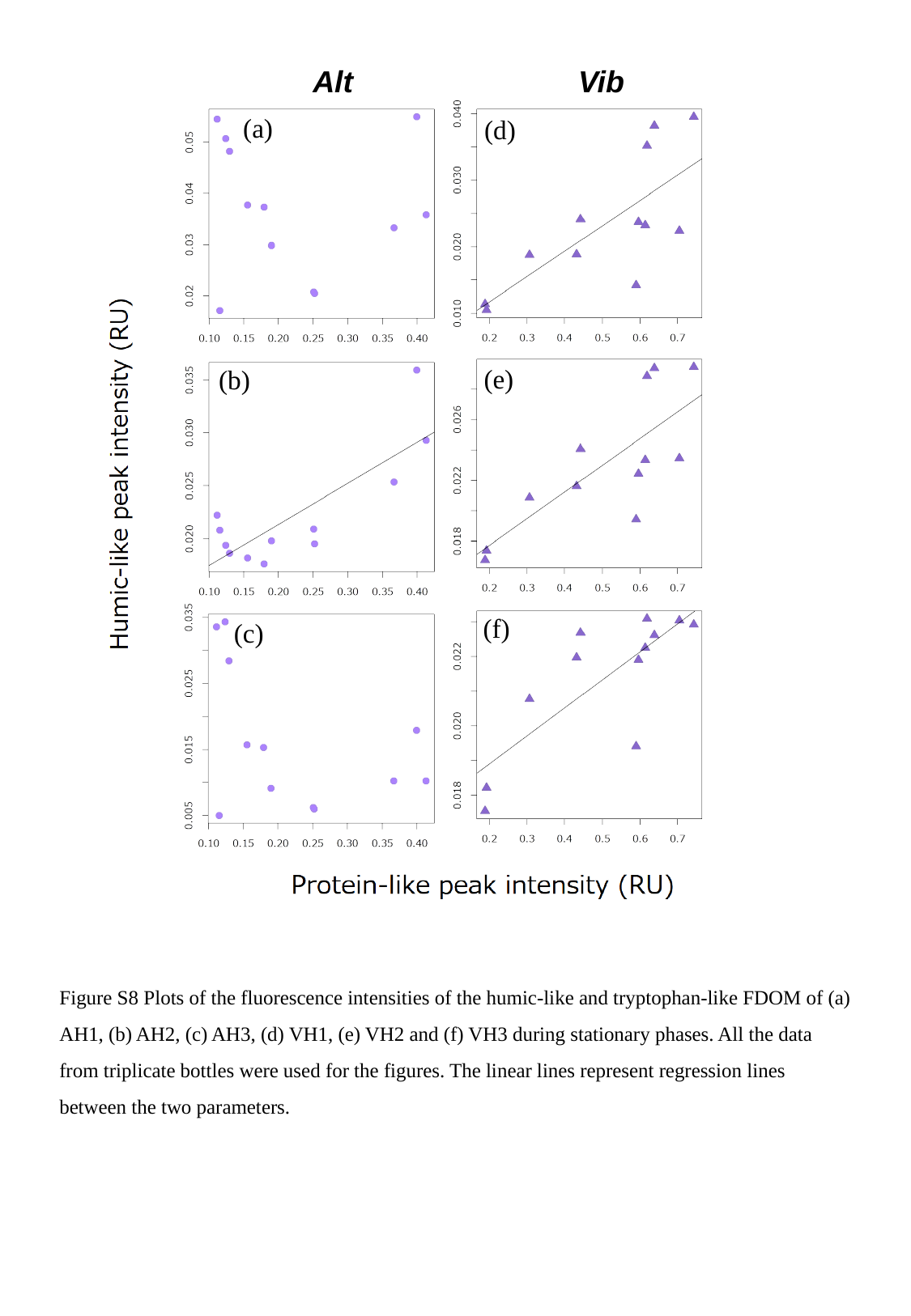

Alt
Vib
(a)
(d)
(e)
(b)
(f)
(c)
Figure S8 Plots of the fluorescence intensities of the humic-like and tryptophan-like FDOM of (a) AH1, (b) AH2, (c) AH3, (d) VH1, (e) VH2 and (f) VH3 during stationary phases. All the data from triplicate bottles were used for the figures. The linear lines represent regression lines between the two parameters.

## Slide 11
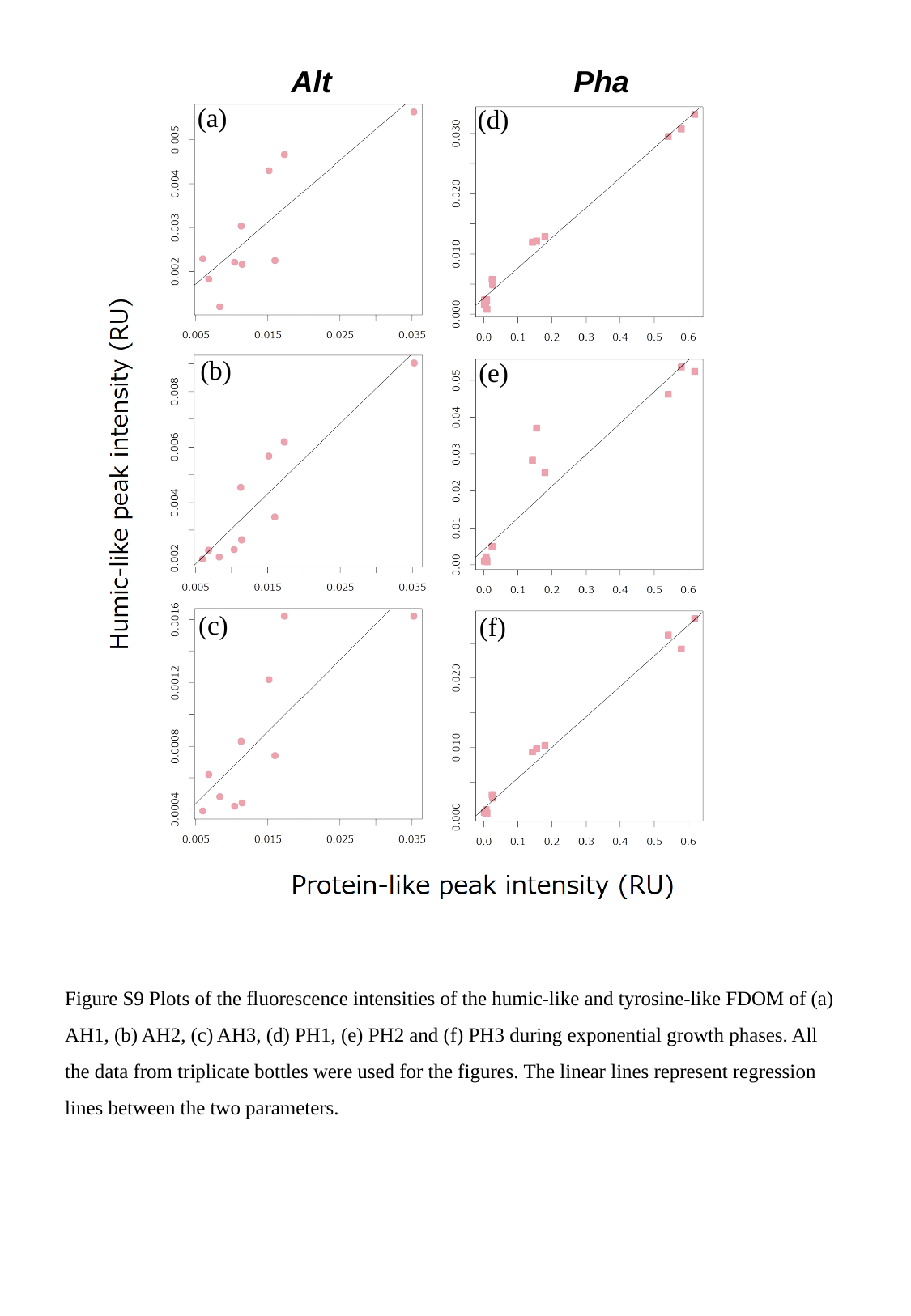

Alt
Pha
(a)
(d)
(b)
(e)
(c)
(f)
Figure S9 Plots of the fluorescence intensities of the humic-like and tyrosine-like FDOM of (a) AH1, (b) AH2, (c) AH3, (d) PH1, (e) PH2 and (f) PH3 during exponential growth phases. All the data from triplicate bottles were used for the figures. The linear lines represent regression lines between the two parameters.

## Slide 12
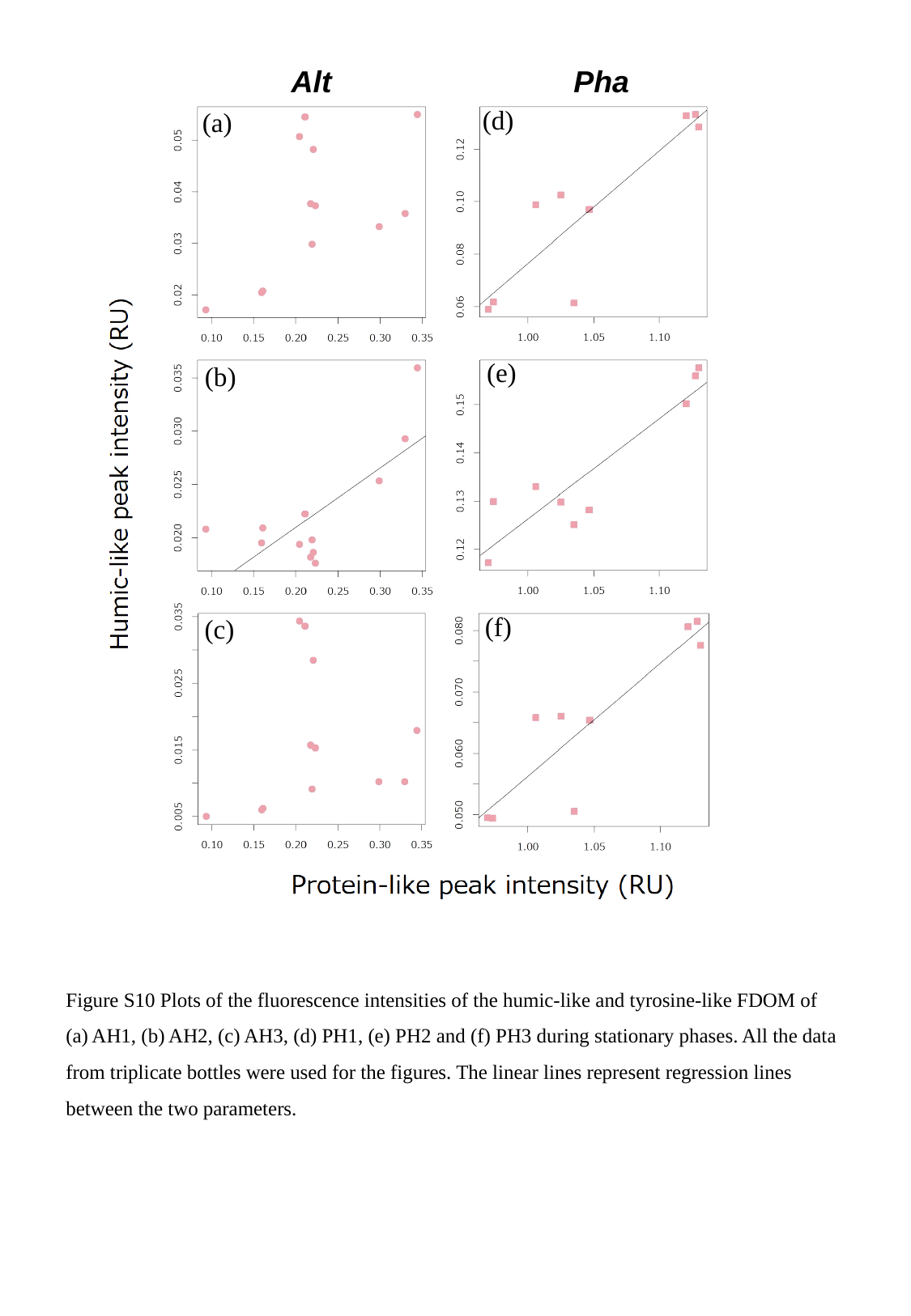

Alt
Pha
(d)
(a)
(e)
(b)
(f)
(c)
Figure S10 Plots of the fluorescence intensities of the humic-like and tyrosine-like FDOM of (a) AH1, (b) AH2, (c) AH3, (d) PH1, (e) PH2 and (f) PH3 during stationary phases. All the data from triplicate bottles were used for the figures. The linear lines represent regression lines between the two parameters.
